# Network hierarchy in Alzheimer’s and Lewy body spectrum disorders: functional gradients, cognitive phenotypes, and translational gaps

**DOI:** 10.3389/fnagi.2026.1896407

**Published:** 2026-08-10

**Authors:** Yujian Liu, Tianjiao Lv, Yuan Li

**Affiliations:** 1 Department of Radiology, Zigong First People’s Hospital, Zigong, China; 2 Department of Ultrasound, Zigong Fourth People’s Hospital, Zigong, China

**Keywords:** Alzheimer’s disease, cortical hierarchy, dementia with Lewy bodies, evidence asymmetry, functional connectivity gradients, Parkinson’s disease dementia, visual hallucinations

## Abstract

Dementia syndromes alter cortical hierarchy in addition to individual brain regions. Functional connectivity gradients offer a continuous, low-dimensional framework for examining these hierarchy-level changes. This focused structured narrative review combines a transparent evidence audit with a candidate-target comparative framework for evaluating gradient evidence in Alzheimer’s disease (AD) and Lewy body spectrum disorders, including dementia with Lewy bodies and Parkinson’s disease dementia. The evidence is asymmetric. AD has comparatively direct evidence for metric-specific change at the default-mode/transmodal end of the principal cortical gradient, whereas Lewy body disease has sparse and partly negative direct functional-gradient evidence. The review defines evidence tiers and eligibility rules for translating conventional functional-connectivity findings into testable gradient hypotheses, and applies them to the Lewy body literature. This framework is intended to improve evidence appraisal when emerging neuroimaging methods are applied to heterogeneous disease populations. Current data support metric-specific principal-gradient change and transmodal dedifferentiation in AD. In Lewy body disease, posterior cortical, attention, salience, and control-network findings remain FC-derived candidate targets for direct testing. The review also addresses gradient-estimation methods, disease-specific confounds, mixed pathology, brain-behavior associations, and validation standards. Gradient measures are not proposed as ready clinical biomarkers. Instead, the review specifies the evidence required to determine whether hierarchy-axis phenotyping adds value beyond conventional connectivity, atrophy, and molecular biomarkers.

## 1 Introduction

Neurodegenerative dementias are increasingly understood as disorders of distributed brain networks rather than purely focal lesions. In Alzheimer’s disease, the earliest and most reproducible systems-level abnormalities involve the default-mode network (DMN) and medial temporal-parietal circuitry. Lewy body disease (LBD) spectrum disorders more prominently involve posterior visual networks, attentional systems, salience circuitry, and frontoparietal control systems (Jack et al., 2018; McKeith et al., 2017). Conventional network analyses have described these abnormalities effectively, but they usually treat networks as discrete modules. This approach is useful for localization but less suited to explaining how disease alters the continuous macroscale hierarchy through which the cortex transforms sensory input into progressively more abstract representations.

For AD, clinical and research frameworks distinguish preclinical disease, mild cognitive impairment (MCI) due to AD, and AD dementia (Sperling et al., 2011; Albert et al., 2011; McKhann et al., 2011). The International Working Group-2 (IWG-2) and later NIA-AA research frameworks moved the field toward biologically anchored classification, a shift that is relevant when evaluating whether gradient metrics add information beyond established biomarkers (Dubois et al., 2014; Jack et al., 2018).

### 1.1 Limitations of conventional functional connectivity frameworks

Resting-state functional MRI has provided a broad set of tools for dementia neuroimaging, including seed-based connectivity, independent component analysis, graph-theoretical measures, dynamic connectivity, and measures of network segregation or integration. These approaches show that AD and LBD are associated with altered large-scale functional connectivity (FC). However, pairwise and network-level summaries do not directly represent a region’s position within a continuous cortical hierarchy. A reduced connection may reflect local disconnection, reduced differentiation, altered network separation, or a shift in a low-dimensional embedding; FC alone cannot distinguish these possibilities. Gradient analysis therefore tests the macroscale organization in which abnormal networks are embedded. This distinction is especially important because both AD and Lewy body disease are biologically heterogeneous (Jack et al., 2018; Scheltens et al., 2021; Aarsland et al., 2021; Walker et al., 2015).

This systems-level perspective is relevant because episodic memory decline, hallucinations, attentional fluctuations, and executive dysfunction arise from failures of coordinated processing across hierarchically organized systems.

### 1.2 Functional gradients and cortical hierarchy

Functional connectivity gradients provide a low-dimensional representation of whole-brain connectivity organization. Margulies et al. (2016) showed that the dominant cortical gradient positions primary sensory and motor systems at one end and transmodal default-mode regions at the other. This principal gradient recapitulates a sensory-to-transmodal hierarchy consistent with older neurobiological accounts of cortical organization, in which information flows from primary sensory cortices to unimodal association areas, heteromodal association cortices, and limbic or paralimbic systems supporting abstract cognition (Mesulam, 1998). Subsequent work has generalized this approach as a macroscale framework for studying cortical organization across development, aging, cognition, and disease (Huntenburg et al., 2018; Vos de Wael et al., 2020). Paquola et al. (2019) and Vázquez-Rodríguez et al. (2019) linked cortical gradients to microstructure and structure-function tethering. Shafiei et al. (2020) emphasized intrinsic cortical dynamics, whereas Gao et al. (2022) showed task-related flexibility of the principal gradient. Recent syntheses and geometric models place these findings within a broader account of cortical organization (Bernhardt et al., 2022; Pang et al., 2023).

In this framework, a gradient is not a new anatomical network but a continuous coordinate system derived from similarity in connectivity profiles. Regions with similar connectivity fingerprints occupy nearby locations in an embedding space, whereas regions with dissimilar connectivity patterns are positioned farther apart. The first gradient commonly captures the sensory-to-transmodal axis; secondary gradients may capture visual-somatomotor separation, task-positive versus task-negative organization, or other axes depending on sample, preprocessing, parcellation, and alignment strategy. Importantly, disease-related changes in gradient structure can be interpreted as changes in cortical hierarchy only when the analytical pipeline and reference framework are explicitly defined.

Earlier gradient reviews established the relevance of macroscale cortical organization for healthy cognition, development, aging, and disease more broadly (Huntenburg et al., 2018; Bernhardt et al., 2022). More recent methodological syntheses emphasize that the wider use of gradient analysis must be accompanied by transparent implementation, shared analytic standards, and explicit limits on biological inference (Royer et al., 2024). The present article is a focused structured narrative review that combines a transparent evidence audit with a candidate-target comparative framework. It asks how direct gradient findings, adjacent disease evidence, and conventional FC results can be compared when the literature is uneven across dementia syndromes.

### 1.3 Evidence asymmetry between AD and Lewy body spectrum disorders

The evidence base is uneven. Direct AD studies span subjective cognitive decline, mild cognitive impairment, and dementia, and report metric-specific principal-gradient or DMN-transmodal changes with cognitive associations. The structured LBD/PDD audit identified one core whole-cortical study showing preliminary structural-gradient reorganization but no significant group-level difference in the first two static functional gradients. It also identified one adjacent striatal-gradient study that included PDD. Accordingly, the LBD/PDD section is an evidence audit and candidate-target synthesis, not a parallel mature gradient review.

The review therefore asks when posterior visual, attention-control, and salience-related FC findings justify a targeted direct-gradient question without being relabeled as gradient abnormalities.

### 1.4 Rationale for an evidence-weighted review framework

This article is not a PRISMA-conformant systematic review or meta-analysis. It is a focused structured narrative review in which an evidence audit makes the inclusion logic and evidentiary boundaries explicit. The audit classifies evidence rather than pooling effects. The analysis addresses three questions: whether AD-related decline is best understood through DMN-transmodal hierarchy changes; whether LBD/PDD FC findings justify specific direct-gradient questions; and whether gradient metrics explain cognitive or behavioral phenotypes beyond conventional FC.

The evidential hierarchy is kept explicit throughout. Direct whole-cortical functional-gradient evidence, direct structural-gradient evidence, adjacent gradient evidence, indirect connectivity evidence, and theoretical predictions are separated in the text, tables, and figure legends. Solid visual elements denote direct functional-gradient evidence, dashed elements denote FC-derived targets requiring direct validation, and dotted elements denote predictions that still lack direct empirical support.

The framework is designed to prevent two errors: treating conventional FC findings as direct gradient evidence and forcing a false symmetry between the AD and LBD/PDD literatures.

### 1.5 Organizational logic: a 2 × N comparative matrix

**FIGURE 1:**
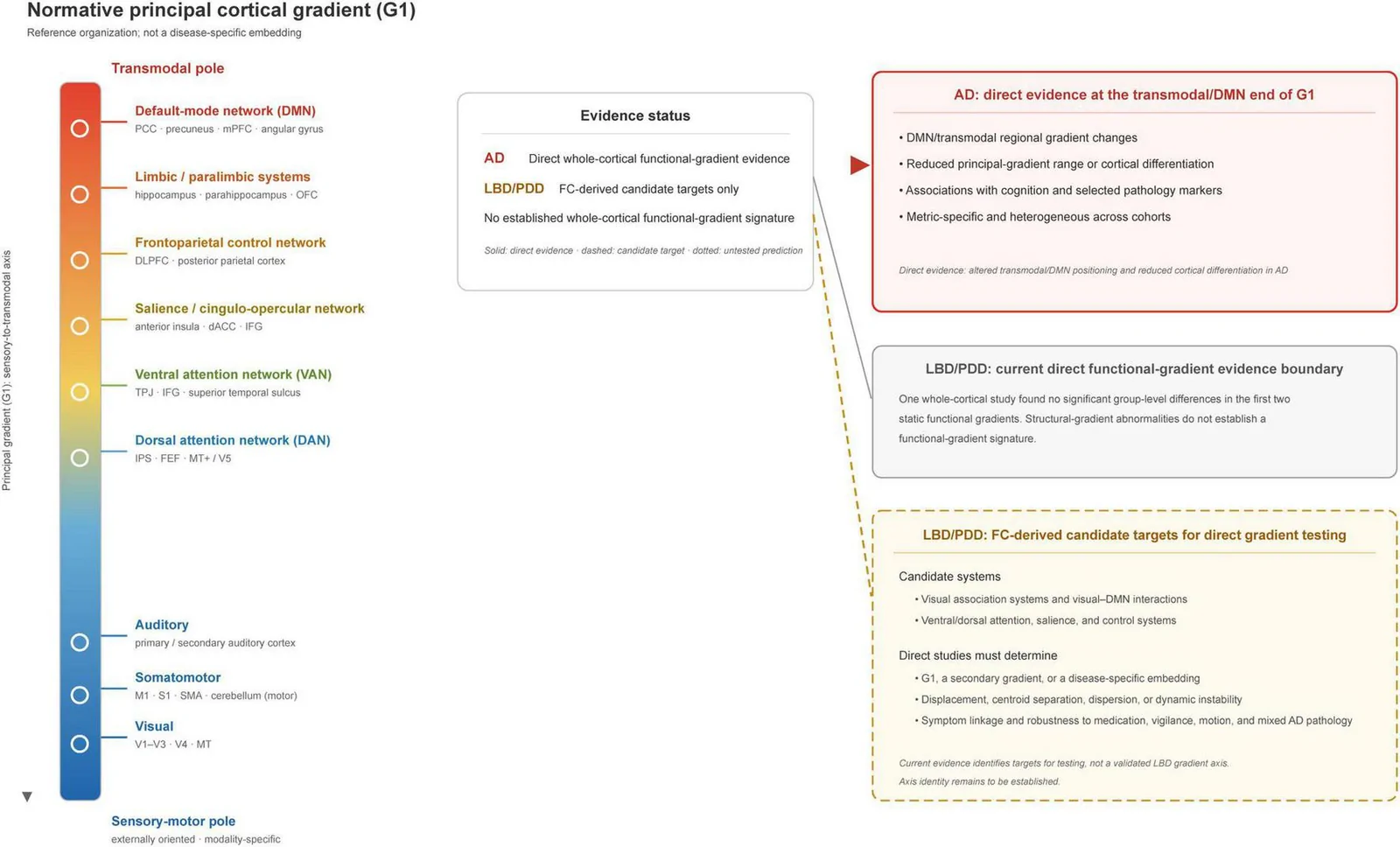
Evidence-weighted hierarchy-axis framework for Alzheimer’s disease and Lewy body spectrum disorders. The normative principal cortical gradient is schematized from sensory–motor systems to transmodal default-mode regions and is shown as a reference organization rather than a disease-specific embedding. In Alzheimer’s disease, solid red elements denote direct whole-cortical functional-gradient evidence implicating metric-specific alteration of transmodal/default-mode organization and cortical differentiation. In Lewy body spectrum disorders, posterior visual, attentional, salience, and control-system findings are represented as functional-connectivity-derived candidate targets for direct gradient testing, rather than as a validated disease-specific functional-gradient axis. The available whole-cortical Lewy body study found no significant group-level differences in the first two static functional gradients; structural-gradient abnormalities therefore do not establish a functional-gradient signature. Dashed elements indicate candidate targets, whereas dotted elements indicate untested axis- or metric-specific predictions. Conceptual sources include Margulies et al. (2016), Mesulam (1998), Huntenburg et al. (2018), and Vos de Wael et al. (2020).

The disease-specific sections first establish the available evidence in AD and LBD. The comparative section then examines shared loss of hierarchical differentiation and divergent candidate axes. The brain-behavior section contrasts phenotype mappings across disorders, and the future-directions section converts weak or unsupported matrix cells into testable research questions. The comparative matrix is summarized in Table 1, whereas Figure 1 provides an evidence-weighted visual overview of the proposed hierarchy-axis framework.

**TABLE 1:** Comparative matrix for AD and Lewy body spectrum disorders.

Dimension	AD spectrum	Lewy body spectrum	Evidence / validation status	Section cross-reference
Dominant hierarchy axis	Principal sensory-to-transmodal/DMN axis.	FC-derived candidate targets in visual, attention, salience, and control systems; no established functional-gradient axis.	AD: Tier A1 direct functional evidence. LBD: Tier C target nomination; available Tier A1 is negative at group level.	6.3
Primary affected systems	DMN, medial temporal, posterior cingulate, angular gyrus.	Visual association, frontoparietal, salience, attention networks.	AD: direct and convergent. LBD: indirect.	4.2, 5.2
Key gradient metric	Metric-specific principal-gradient compression and reduced DMN–transmodal extremity.	No validated metric. Direct studies must pre-specify regional displacement, centroid separation, compression, or temporal instability.	AD: published Tier A1 metrics. LBD: no replicated Tier A1 functional metric.	4.2, 5.6
Key cognitive–behavioral phenotype	Episodic memory decline; internally directed cognition.	Visual hallucinations; cognitive fluctuation; visuospatial impairment.	AD: gradient–behavior evidence emerging. LBD: FC–behavior evidence only.	7.1–7.4
Mixed pathology prediction	AD component should be operationalized by amyloid- and tau-positive biomarker status.	Mixed group: probable DLB/PDD with dopaminergic support plus amyloid and tau positivity; autopsy remains reference standard.	Untested; requires pre-specified biomarker strata, continuous pathology measures, and pathology anchoring.	6.4, 9.2
Current validation status	Direct group-level evidence; no replicated individual-level classifier, external validation, or clinical-utility analysis.	No replicated direct functional-gradient signature or individual-level validation.	Both remain research-stage; readiness requires reliability, calibration, external validation, incremental value, and clinical net benefit.	6.5, 8.2–8.4

AD-related gradient claims are treated as direct where supported by primary Tier A1 studies. Lewy body spectrum claims are treated as candidate targets unless supported by primary Tier A1 evidence. Tier A2 structural-gradient findings are reported separately and do not substitute for a functional-gradient conclusion. Current validation status is defined by observable milestones: reliability, calibration, external validation, incremental performance beyond established measures, and evidence of clinical net benefit. In figures, solid elements denote direct functional-gradient evidence, dashed elements denote FC-derived targets requiring direct validation, and dotted elements denote predictions without direct empirical support. AD, Alzheimer’s disease; LBD, Lewy body disease; DMN, default-mode network; FC, functional connectivity.

## 2 Conceptual basis: functional gradients and cortical hierarchy

### 2.1 Principal gradients and sensory-to-transmodal organization

The principal gradient of cortical functional organization provides a compact description of the transition from externally oriented, modality-specific processing to internally oriented, integrative cognition. At one pole are primary and unimodal sensory-motor systems that process immediate environmental information. At the opposite pole are transmodal association regions, particularly components of the DMN such as posterior cingulate cortex, precuneus, angular gyrus, and medial prefrontal cortex. These transmodal regions are late-myelinating, phylogenetically expanded, densely connected to association systems, and repeatedly implicated in network-based models of neurodegenerative vulnerability (Huntenburg et al., 2018; Margulies et al., 2016; Seeley et al., 2009). Contemporary accounts of the DMN support its role in internally oriented cognition (Buckner and DiNicola, 2019; Smallwood et al., 2021). Attention and salience-control models provide the complementary language for intermediate systems that regulate selection, switching, and control (Menon and Uddin, 2010; Petersen and Posner, 2012).

The principal-gradient framework complements established network labels by embedding them in a continuous hierarchy. The DMN is both a named network and the transmodal extreme of a cortical manifold. Visual and somatomotor systems anchor lower-order portions of large-scale organization, while frontoparietal, salience, dorsal attention, and ventral attention systems occupy intermediate or axis-specific positions that mediate perception, action, attentional selection, and abstract cognition.

Gradient organization also extends beyond the isocortex. Katsumi et al. (2023) showed that functional connectivity gradients of the isocortex, cerebellum, and hippocampus can be related within a common systems-level framework, using large discovery and validation samples. This finding supports the use of gradient thinking for cross-structure hierarchy models, while keeping non-cortical gradient results conceptually separate from whole-cortical principal-gradient evidence.

### 2.2 Gradient metrics and terminology

Several gradient-derived metrics are relevant to dementia research. Parcel-level gradient scores describe the position of a cortical parcel along an aligned gradient axis, whereas network-level scores summarize the position of a large-scale system. Gradient dispersion denotes the spread of parcels or networks in gradient space. Global axis compression denotes a directly measured reduction in gradient range or in separation between estimated gradient extremes. Regional displacement denotes a directly measured shift in aligned regional or network coordinates. Between-network boundary blurring denotes a directly measured reduction in centroid separation or an increase in overlap between network distributions. Gradient alignment, often performed by Procrustes rotation to a group or template gradient, is required because gradient polarity and component order are otherwise arbitrary (Vos de Wael et al., 2020).

These terms are not interchangeable. A regional score shift is not global axis compression, and reduced within-network dispersion is not reduced between-network separation. A second gradient should not be labeled as visual-attention-control unless its anatomical and functional anchors are characterized in the study sample. In this review, dedifferentiation denotes an interpretation supported by directly measured loss of separation, range, or network specificity. “Hierarchy disruption” is used only as an umbrella term for one or more directly estimated, metric-specific changes; it is never inferred from FC alone. Informative studies should report the parcellation scheme, affinity kernel, embedding algorithm, retained components, alignment strategy, and exact metric definitions.

### 2.3 Cortical hierarchy, network segregation, and dedifferentiation

Cortical hierarchy is maintained by both segregation and integration. Segregation allows sensory, attentional, control, and transmodal systems to preserve distinct functional profiles, while integration permits communication across levels of abstraction. In this review, dedifferentiation refers to a metric-supported loss of that normal separation; it is not a synonym for any regional gradient-score difference. Depending on the direct metric, it may involve lower global axis range, reduced separation between sensory and transmodal poles, reduced between-network dispersion or centroid distance, or displacement toward a less distinctive position in gradient space.

Healthy aging is associated with changes in functional-gradient organization, including altered dispersion and reduced differentiation in some systems (Bethlehem et al., 2020). Neurodegenerative disease may accelerate, spatially bias, or qualitatively alter this process. Dedifferentiation, however, is not automatically pathological in every setting. Some reorganization may reflect compensation, disease-stage-specific adaptation, or methodological artifact. A defensible dementia-gradient framework therefore tests whether gradient changes relate to cognition, behavior, pathology, or progression beyond structural atrophy alone.

### 2.4 Why neurodegenerative diseases may preferentially disrupt hierarchy axes

Three mechanisms motivate a hierarchy-axis account of neurodegeneration. First, hub vulnerability suggests that highly connected association regions may face greater synaptic activity and greater exposure to network-mediated pathology. The networks targeted by different neurodegenerative diseases overlap with intrinsic connectivity patterns in the healthy brain, consistent with disease propagation or expression along pre-existing network architecture (Seeley et al., 2009; Zhou et al., 2012). Second, epicenter-propagation models propose that pathological proteins spread along structural or functional connectome edges. In AD, this mechanism is relevant because transmodal association regions occupy extreme positions on the principal gradient and are vulnerable to medial temporal-parietal network pathology. Network-based tau-spread models provide complementary support for this account (Ottoy et al., 2024; Vogel et al., 2020). In Lewy body disease, α-synuclein pathology involves brainstem, limbic, and cortical systems; whether cortical gradient axes capture its cognitive and behavioral expression remains unknown. Third, transmodal and association systems have high metabolic and integrative demands and overlap with axes of transcriptomic specialization (Burt et al., 2018). These features may increase vulnerability to mitochondrial dysfunction, synaptic failure, neurotransmitter imbalance, and multimodal pathology.

These mechanisms do not predict a universal disease axis. They motivate disease-specific tests, while evidence grading distinguishes direct observations from candidate targets.

## 3 Methodological framework for gradient-based dementia research

This section defines the methodological terms needed for the disease-specific synthesis. It precedes the evidence review because apparent disagreement between studies may arise from analytic differences rather than biology. Key theoretical and methodological literature informing these analytic choices is summarized in Table 2.

**TABLE 2:** Key theoretical and methodological literature.

References/concept	Core contribution	Relevance to this review	Primary section
Mesulam (1998)/cortical hierarchy	Provides a classical account of sensory-to-association cortical organization.	Defines the conceptual basis for interpreting dementia as disruption of hierarchical processing rather than isolated network failure.	Section 2
Margulies et al. (2016)/principal gradient	Introduces the dominant sensory-to-transmodal gradient of macroscale cortical organization.	Provides the coordinate system used to interpret AD as DMN–transmodal hierarchy disruption.	Sections 2 and 4
Huntenburg et al. (2018)/large-scale gradients	Synthesizes gradients as organizing principles of cortical function.	Places discrete networks within continuous hierarchy axes.	Section 2
Katsumi et al. (2023)/cross-structure gradients	Shows correspondence among isocortical, cerebellar, and hippocampal functional connectivity gradients.	Supports cross-structure hierarchy thinking while preserving the distinction between cortical and noncortical gradient evidence.	Sections 2 and 4
Vos de Wael et al. (2020)/BrainSpace	Provides a widely used toolbox for gradient estimation, alignment, and visualization.	Anchors the methodological discussion of diffusion embedding, alignment, and comparability.	Section 3
Bethlehem et al. (2020)/gradient dispersion across lifespan	Shows that gradient dispersion varies across adult life.	Frames aging and disease-related dedifferentiation as related but non-identical processes.	Sections 2 and 3
Watson and Andrews (2023)/methodological caution	Highlights interpretational limitations of connectopic and gradient-like mapping.	Provides a safeguard against overinterpreting FC-derived candidate targets, especially in LBD/PDD.	Sections 3 and 5
Royer et al. (2024)/gradient-methods implementation	Synthesizes methodological maturation, open workflows, and community standards for brain-gradient research.	Supports the requirement for transparent implementation and explicit interpretational limits when translating gradients to dementia research.	Sections 1 and 3
Markello et al. (2022)/neuromaps	Provides standardized coordinate transformation, reference-map annotation, and spatial-autocorrelation-preserving null models.	Supports reproducible map comparison and spatial inference; it does not replace pre-specification of the gradient metric or biological claim.	Section 3
Bouzigues et al. (2025)/FTD comparator	Reports metric-specific functional-gradient disruption in frontotemporal dementia beyond the distribution of atrophy.	Serves as an external neurodegeneration comparator; it supports the general plausibility of hierarchy reorganization but is not direct AD or LBD evidence.	Section 4.5

This table serves as a conceptual and methodological reading map for sections 2 and 3; it is not a systematic evidence synthesis. AD, Alzheimer’s disease; DMN, default-mode network; FC, functional connectivity; FTD, frontotemporal dementia; LBD/PDD, Lewy body disease/Parkinson’s disease dementia.

### 3.1 Resting-state fMRI preprocessing and parcellation strategies

Gradient estimates depend on transparent resting-state fMRI preprocessing. Head motion, physiological noise, global signal regression, temporal filtering, spatial smoothing, and nuisance regression all influence FC matrices. In dementia cohorts, motion may also covary with disease severity, sleepiness, parkinsonism, and fluctuation. Studies should therefore report framewise displacement, scrubbing, nuisance-regression choices, and sensitivity analyses (Power et al., 2012, 2014; Ciric et al., 2017; Parkes et al., 2018; Murphy and Fox, 2017; Esteban et al., 2019).

Parcellation affects both gradient stability and anatomical interpretation. Schaefer, Glasser, Yeo, disease-specific, and subcortical atlases differ in resolution and biological assumptions (Yeo et al., 2011; Glasser et al., 2016; Schaefer et al., 2018; Van Essen et al., 2013). Fine parcellations increase spatial detail but can increase noise. Coarse parcellations are more stable but may obscure regional displacement. Cross-study synthesis therefore requires explicit atlas reporting and, where possible, atlas-sensitivity analyses. Surface reconstruction and parcellation workflows, including FreeSurfer-based pipelines, can also influence downstream gradient construction (Fischl, 2012).

### 3.2 Functional connectivity matrices and affinity mapping

Most pipelines derive a Fisher-transformed FC matrix and then construct an affinity matrix describing similarity between regional connectivity profiles. Cosine similarity, normalized angle, sparsification, and treatment of negative correlations determine which connectivity features dominate the embedding and can materially change the resulting manifold. These steps must therefore be reported before biological interpretation.

### 3.3 Diffusion map embedding and manifold learning

Diffusion map embedding is widely used for functional-gradient estimation because it captures nonlinear relations among connectivity profiles while preserving manifold geometry (Coifman and Lafon, 2006). Component ordering and interpretation may nevertheless vary with the embedding method, retained components, diffusion parameters, and the use of static or dynamic FC. These choices are especially consequential in fluctuating syndromes (Allen et al., 2014; Preti et al., 2017; Lurie et al., 2020; Brown et al., 2022; Vidaurre et al., 2017).

In dementia research, the first two or three gradients are usually the most interpretable. Gradient numbering, however, does not always reproduce canonical patterns. A component appearing first in one sample may appear second in another because of cohort characteristics, preprocessing, parcellation, or explained variance. Anatomical anchors such as sensory-to-transmodal or visual-somatomotor are therefore more informative than component number alone.

### 3.4 Gradient alignment, compression, and dispersion metrics

Gradient alignment is mandatory for group comparison because eigenvectors have arbitrary sign and may rotate within low-dimensional space. Procrustes alignment to a group or external template improves comparability (Vos de Wael et al., 2020); unaligned individual or group gradients require cautious interpretation.

Compression and dispersion demand metric-level precision. Global axis compression should be judged by a direct reduction in range or extreme-to-extreme separation. Network-specific dispersion describes spread within a system, whereas boundary blurring requires direct evidence of reduced between-network separation or greater overlap. In disease cohorts, these metrics are most informative when tested against cognitive, behavioral, structural, or molecular markers rather than reported only as case-control differences.

### 3.5 Sources of methodological heterogeneity

Methodological heterogeneity arises from scanner field strength, acquisition duration, wakefulness, disease stage, medication status, parcellation, global signal regression, motion thresholds, affinity kernel, embedding algorithm, template choice, and statistical correction. These factors limit comparisons of AD effect sizes. They are particularly consequential in LBD/PDD, where small samples, diagnostic uncertainty, fluctuation, parkinsonism-related motion, and hallucination status can reduce reproducibility.

LBD/PDD cohorts introduce additional sources of bias. Fluctuating alertness, episodic hallucinations, cholinesterase inhibitors, dopaminergic or antipsychotic exposure, sleepiness, parkinsonism, and visual impairment can all influence network estimates. Future studies should report medication state, symptom timing, vigilance, visual function, scan duration, and motion-quality metrics.

Five operational evidence tiers are used. Tier A1 denotes direct whole-cortical functional-gradient evidence in the target disease group, including case-control, symptom-gradient, and pathology-gradient analyses. Tier A2 denotes direct whole-cortical structural-gradient evidence in the target disease group. It may support structural hierarchy reorganization but cannot substitute for a functional-gradient conclusion. Tier B denotes adjacent gradient evidence from a related population or anatomical system, including PD-only whole-cortical studies and striatal gradients in PD/PDD. Tier C denotes indirect functional, structural, electrophysiological, or metabolic connectivity evidence. Such evidence can nominate, but not establish, a target for direct gradient testing. Tier D denotes an untested theoretical prediction. A single multimodal study may contribute to more than one tier, but a Tier A2 result never upgrades a missing or negative Tier A1 result.

### 3.6 Reproducibility challenges and interpretational limitations

Large multisite reproducibility studies of dementia gradients are still lacking. Formal meta-analysis is constrained by heterogeneous pipelines, populations, and metrics. This limitation is particularly important in LBD/PDD, where direct group-level functional evidence remains limited.

A further question is whether gradients add information beyond the FC matrix. Watson and Andrews (2023) showed that connectopic or gradient-like maps can be influenced by spatial autocorrelation, smoothing, interpolation, and pipeline-specific features. They therefore cautioned against treating gradients as self-evident biological axes. Spatial-null-model work likewise shows that brain-map associations can be inflated when spatial autocorrelation is ignored (Burt et al., 2020; Markello and Misic, 2021). Tools such as neuromaps can standardize coordinate transformation, reference-map annotation, and spatial-null testing, but they do not remove the need to pre-specify the gradient metric and inferential target (Markello et al., 2022). These concerns call for stricter interpretation, not abandonment, of whole-brain gradient analysis. Gradient claims are most persuasive when they show incremental value beyond conventional FC, relate to behavior or pathology, and remain robust to transparent alignment, parcellation, and motion-control procedures. Supplementary Table 1 documents methodological and validation heterogeneity across the AD studies. This rationale underpins the incremental-validity test in section 9.3.

### 3.7 Scope, evidence identification, and study classification

This manuscript is a focused structured narrative review with a transparent evidence audit and a candidate-target comparative framework. It was not designed as a PRISMA-conformant systematic review or meta-analysis. The audit evaluates the central evidence-asymmetry claim by distinguishing findings that directly estimate functional gradients from adjacent evidence and from studies that only nominate targets for future direct analysis.

PubMed/MEDLINE was searched from database inception to 30 April 2026. Google Scholar, forward and backward citation chaining, and recent reviews were used to identify additional eligible reports. Search strings paired AD, MCI, amyloid-risk, LBD, DLB, PDD, PD, and related cognitive terms with functional, structural, connectome, principal, cortical, hierarchical, manifold, and gradient terms. Complete search strings, eligibility criteria, and operational evidence-tier definitions are provided in Supplementary Table 2. Eligible direct studies were peer-reviewed original human studies that estimated a gradient, manifold, or comparable low-dimensional connectivity axis in the relevant disease population. Adjacent studies examined a relevant but non-equivalent population or anatomical system. Conventional FC, EEG, structural-connectivity, and metabolic-connectivity studies were retained as Tier C evidence only when they were phenotype-linked or sufficiently network-specific to nominate a candidate direct-gradient target. Reviews, conference abstracts, preprints, non-human studies, and studies without original eligible imaging data were excluded from the structured evidence tables. Within this focused audit, no additional eligible peer-reviewed Tier A1 DLB/PDD report was identified. The final structured synthesis includes nine AD gradient studies (Table 3) and fifteen LBD/PD evidence reports (Table 4). Supplementary Figure 1 shows the retained evidence sets and their operational classification.

**TABLE 3:** AD spectrum gradient evidence: study-specific synthesis.

Study	Cohort/modality	Gradient target	Main finding	Use in this review	Quality indicator
Hu et al. (2022)	49 HC, 49 MCI, 49 AD; rs-fMRI	Whole-brain connectome gradients (G1, G2)	Posterior DMN secondary-gradient values decreased in AD; anterior–posterior DMN imbalance across MCI and AD. Disease-sensitive DMN effect expressed in secondary gradient.	Direct DMN-anchored hierarchy evidence; not used as canonical G1 compression evidence because component ordering differs across pipelines.	Balanced 49/49/49 cohort; gradient–cognition links; component-ordering caution.
He et al. (2023)	aMCI, AD, controls; rs-fMRI	Whole-brain functional gradients and gradient dispersion	Unimodal-to-transmodal gradient suppression more distributed in AD than aMCI; global and module-level dispersion reduced in AD; dispersion changes associated with cognitive scores.	Supports progressive cortical dedifferentiation from aMCI to AD.	Group/dispersion contrasts and cognitive links; pipeline heterogeneity limits comparison.
Wang et al. (2024)	809 samples from seven scanners; rs-fMRI	Principal primary-to-transmodal gradient; regional and global gradient metrics	AD showed reduced gradient range and variation, increased scores in somatomotor/ventral attention/frontoparietal regions, and decreased scores in DMN.	Multi-center support for AD-related principal-gradient suppression and DMN–transmodal disruption.	Large multi-scanner cohort (*n* = 809); robust directionality; standardized effect size not uniformly reported.
Liu et al. (2025)	45 Controls, 45 MCI, 45 AD; rs-fMRI	Hemispheric functional-gradient lateralization	AD showed reduced interhemispheric gradient lateralization, most pronounced in the DMN and control networks; DMN lateralization correlated with cognition.	Extends AD gradient evidence to hemispheric asymmetry; not an individual biomarker.	Balanced three-group design; cognition link; needs replication.
Ottoy et al. (2024)	TRIAD cohort; tau-PET, amyloid-PET, MRI, dMRI, fMRI	Functional and structural gradients plus molecular PET gradients	Functional/structural gradients altered in AD; tau distribution followed principal axes of brain organization; gradient contraction related to greater tau burden.	Mechanistic bridge between connectome hierarchy, tau pathology, and cognition.	Multimodal PET/MRI/dMRI; pathology–gradient coupling, not simple group effect.
He et al. (2024)	188 Cognitively unimpaired older adults with/without amyloid positivity; fMRI and spontaneous speech	Large-scale cortical hierarchy / functional gradients	Amyloid-positive individuals showed altered cortical hierarchies and reduced gradient variability; gradient variability related to prosodic speech measures and digit span.	Supports preclinical hierarchy alteration and links gradient measures to subtle behavioral readouts.	Preclinical Aβ-positive cohort; speech/cognition links; replication needed.
Song et al. (2025)	271 Participants stratified by AD diagnosis and APOE ε4; rs-fMRI and plasma biomarkers	DMN functional gradients using BrainSpace	Group-by-genotype interaction in right inferior temporal gyrus; AD APOE ε4 carriers showed marked gradient attenuation; gradient related to cognition in carriers; LSFG–RITG connectivity mediated amyloid effect.	Genotype-specific evidence for DMN hierarchy vulnerability; requires replication before biomarker claims.	Genotype interaction with cognition/biomarker mediation; hypothesis-generating.
Tan et al. (2025)	AD-spectrum cohort; functional and structural connectome gradients	Entorhinal stepwise connectivity projected into gradient space	Entorhinal connectivity changes characterized across multi-step functional/structural gradients, linking early medial temporal vulnerability to distributed hierarchy organization.	Anatomical mechanism linking entorhinal vulnerability, connectivity propagation, and cortical hierarchy.	Topology/projection evidence; not whole-cortical group-effect.
Yao et al. (2025)	ADNI-3: 58 AD, 103 MCI, 85 controls; MRI	Cerebello-thalamo-cortical connectivity and thalamic/cerebellar gradients	AD/MCI showed CTC hyperconnectivity and thalamic/cerebellar gradient disruptions; classification was strongest for AD versus controls.	Extends gradient analysis beyond the cortex to subcortical and cerebellar systems; not treated as whole-cortical principal-gradient evidence.	Subcortical/CTC extension; not whole-cortical principal-gradient evidence.

AD, Alzheimer’s disease; MCI, mild cognitive impairment; HC, healthy controls; aMCI, amnestic mild cognitive impairment; rs-fMRI, resting-state functional MRI; DMN, default-mode network; G1, first gradient component; G2, second gradient component; dMRI, diffusion MRI; PET, positron emission tomography; CTC, cerebello-thalamo-cortical; APOE, apolipoprotein E; ADNI, Alzheimer’s Disease Neuroimaging Initiative. LSFG, left superior frontal gyrus; RITG, right inferior temporal gyrus.

**TABLE 4:** Audit of direct and adjacent gradient studies in Lewy body spectrum disorders.

Tier	Study	Population/method	Main finding	Symptom link	Criteria status	Permissible use
A1 + A2	Zarkali et al. (2025)	LBD/PD-NC/controls; whole-cortical structural and functional gradients	Structural-gradient reorganization in LBD; functional G1/G2 group differences not significant.	Structural composite score related to cognitive severity.	A1: direct functional result negative; A2: preliminary structural result positive.	Use as the primary boundary condition: structural findings remain A2; static whole-cortical functional G1/G2 result is A1 negative.
B	Wu et al. (2024)	PD patients and controls; whole-cortical functional and structural gradients	Atypical cortical hierarchy in PD involving sensorimotor/executive systems.	Motor/treatment-related links.	Adjacent whole-cortical PD gradient evidence.	PD-spectrum context for cortical-gradient susceptibility only; not LBD proof.
B	Orlando et al. (2025)	PD cohort studied on/off dopamine; whole-cortical functional gradients	Dopamine medication state modulated functional-gradient organization in PD.	Medication-state relevance only.	Adjacent PD evidence; highlights medication confounding.	Justify medication reporting and caution in LBD/PDD gradient design.
B	Li et al. (2025)	PD cognitive spectrum including PDD; striatal functional gradients	Cognition-related striatal-gradient organization; no whole-cortical hierarchy claim.	Cognition links (MoCA, executive).	Adjacent subcortical gradient evidence only.	PD cognitive-spectrum context; not direct LBD/PDD cortical evidence.
C	Lowther et al. (2014)	15 DLB, 13 AD, 40 controls; rs-fMRI ICA	Reduced DMN, salience, and executive connectivity; greater basal-ganglia/limbic coupling.	UPDRS and fluctuation links.	Broad indirect FC evidence; spatially diffuse.	DLB network vulnerability background; not single-axis proof.
C	Schumacher et al. (2018)	31 DLB, 29 AD, 31 controls; rs-fMRI ICA/FSLNets	Reduced within-network FC in motor, temporal, and frontal networks; between-network FC largely preserved.	No significant clinical links identified.	Indirect FC evidence.	Cautionary background evidence.
C	Peraza et al. (2014)	16 Fluctuating DLB, 17 controls; rs-fMRI ICA/dual regression	Reduced left frontoparietal, temporal, and sensorimotor FC; DMN relatively intact.	Left frontoparietal desynchronization associated with fluctuation severity and frequency.	Symptom-linked indirect FC evidence.	Support salience/frontoparietal fluctuation candidate target.
C	Mehraram et al. (2022)	25 Hallucinating vs. 17 non-hallucinating LBD; EEG source connectivity + DTI	Weakened visual ventral connectivity and visual–attention/DMN coupling; occipital lobe most disconnected.	Direct hallucination contrast; NBM/thalamic structural links.	Strong symptom-specific indirect evidence.	High-priority support for visual–attention hallucination candidate target.
C	Firbank et al. (2024)	41 LBD with hallucinations, 48 without, 60 controls; rs-fMRI	Reduced VAN and visual–DMN connectivity in hallucinating LBD.	Visual–DMN link to pareidolia; VAN to visuospatial performance.	Strong symptom-specific FC evidence.	High-priority support for visual–DMN/VAN candidate target.
C	Pezzoli et al. (2021)	DLB with vs. without visual hallucinations; seed-based/ICA rs-fMRI	Altered thalamic, parietal, putaminal, visual, and DMN connectivity.	Hallucination contrast.	Preliminary symptom-specific FC evidence.	Support hallucination candidate target.
C	Zorzi et al. (2021)	26 probable DLB; FDG-PET/MR metabolic connectivity	Altered visual, dorsal-attention, DMN, insular, and orbitofrontal metabolic connectivity.	Hallucination severity correlation.	Cross-modal indirect evidence.	Support hallucination phenotype.
C	Gabriel et al. (2025)	90 DLB, 25 AD, 34 controls; rs-fMRI ROI-to-ROI	Reduced salience and frontoparietal connectivity, strongest at dementia stage.	Fluctuation, RBD, and cognitive decline links.	Strong symptom-linked FC evidence.	Support salience/frontoparietal progression and fluctuation candidate target.
C	Schumacher et al. (2021)	31 MCI-LB, 28 MCI-AD, 24 controls; static/dynamic rs-fMRI	No significant static or dynamic FC differences versus controls or MCI-AD.	No gradient-relevant symptom link identified.	Negative prodromal FC evidence.	Caution against early LBD sensitivity claims.
C	Nicastro et al. (2021)	DLB vs controls; structural connectome	Structural module disorganization involving DMN and dorsal attention networks.	No direct FC–gradient symptom link.	Structural adjacent evidence.	Structural connectivity background only.
C	Kucikova et al. (2024)	Systematic review of fMRI/EEG resting-state connectivity in DLB	DLB resting-state connectivity–symptom links are heterogeneous and method-dependent.	Review-level synthesis only.	Heterogeneity context evidence.	Methods limitations and heterogeneity context.

Tier A1 = direct whole-cortical functional-gradient evidence in the target disease group; Tier A2 = direct whole-cortical structural-gradient evidence in the target disease group; Tier B = adjacent gradient evidence from a related population or anatomical system; Tier C = indirect FC, structural-connectivity, EEG, or metabolic-connectivity evidence; Tier D = untested theoretical prediction. The same multimodal study may contribute to A1 and A2. A2 evidence does not substitute for a functional-gradient conclusion, and Tier C evidence can nominate but cannot establish a gradient effect. LBD, Lewy body disease; PD-NC, Parkinson’s disease with normal cognition; DLB, dementia with Lewy bodies; PDD, Parkinson’s disease dementia; FC, functional connectivity; rs-fMRI, resting-state functional MRI; ICA, independent component analysis; DTI, diffusion tensor imaging; VAN, ventral attention network; DMN, default-mode network; NBM, nucleus basalis of Meynert; UPDRS, Unified Parkinson’s Disease Rating Scale; RBD, REM sleep behavior disorder; MCI-LB, mild cognitive impairment with Lewy bodies; EEG, electroencephalography. Three constraints follow from the audit: (1) LBD/PDD does not yet have established functional-gradient disruption; (2) striatal PDD gradients remain separate from whole-cortical hierarchy gradients; (3) FC-derived posterior attention-control targets must be labeled as indirect and tested with a pre-specified direct gradient metric.

## 4 Alzheimer’s disease spectrum: direct but heterogeneous evidence for DMN–transmodal gradient disruption

Across resting-state fMRI, structural MRI-derived network gradients, and multimodal PET-MRI cohorts, AD studies report metric-specific changes in sensory-to-transmodal organization, DMN-associated position, and functional segregation. The literature is heterogeneous, so the following sections distinguish group-level gradient metrics from behavioral associations and mechanistic links to amyloid, tau, APOE ε4, and structural network vulnerability. Changes in gradient range, regional position, network separation, and lateralization are not treated as interchangeable or as a single uniform AD phenotype. The direct and related AD-spectrum studies informing this synthesis are summarized in Table 3, with their evidence scope, cohort characteristics, gradient metrics, and reported cognitive or biological associations.

### 4.1 Network vulnerability across the AD continuum

Early AD studies suggest that disease can alter the position of large-scale systems within cortical hierarchy space rather than merely reduce connectivity within isolated nodes. Hu et al. examined 49 healthy controls, 49 patients with mild cognitive impairment, and 49 patients with AD using resting-state connectome-gradient mapping. The disease-sensitive DMN effect appeared in a secondary gradient: posterior DMN values were lower in AD than in controls, with a broad anterior-posterior DMN imbalance across MCI and AD. We interpret this as DMN-anchored hierarchy evidence rather than canonical principal-gradient compression because component order can vary across samples and pipelines. As noted in section 3.3, anatomical anchors are more informative than gradient number alone. The same study related altered regional gradient values to logical memory and dementia severity (Hu et al., 2022).

Subsequent work extended this observation from network-level shifts to broader functional segregation. He et al. studied amnestic MCI (aMCI) and AD using functional-gradient mapping and gradient dispersion. They reported a broader reduction in first-gradient scores in AD than in amnestic MCI, together with lower global and module-level dispersion in AD and more limited change in aMCI. Lower dispersion was associated with cognitive scores, supporting an association between progressive loss of functional segregation and cognitive decline rather than diagnostic labels alone (He et al., 2023).

### 4.2 Gradient compression and cortical dedifferentiation

One of the clearest group-level AD findings is a reduced range and variation of the principal sensory-to-transmodal gradient. In a multi-scanner Human Brain Mapping study, Wang et al. reported lower gradient range and variation in AD, higher scores in somatomotor, ventral-attention, and frontoparietal regions, and lower scores in the DMN (Wang et al., 2024). These directly measured changes are compatible with reduced differentiation, provided that this interpretation remains tied to the reported metrics.

This multi-center study extends evidence beyond a single cohort. Reduced range, lower variation, and regional score shifts are related but not interchangeable; accordingly, “compression” is reserved for reduced range or extreme separation.

Recent AD work further suggests that gradient-based lateralization may change across disease stages. Liu et al. (2025) studied 45 controls, 45 patients with MCI, and 45 patients with AD using functional-gradient lateralization metrics. AD patients showed lower interhemispheric lateralization in default-mode and control networks, and DMN lateralization was positively associated with cognition. This extends the literature beyond global axis-range measures, but it remains an emerging group-level marker rather than an individual diagnostic biomarker.

### 4.3 Amyloid, tau, APOE, and connectome-gradient vulnerability

The AD gradient literature is more informative when functional hierarchy is linked to molecular pathology. Ottoy et al. used the multimodal TRIAD cohort, which included tau-PET, amyloid-PET, structural MRI, diffusion MRI, resting-state fMRI, and cognitive testing. They reported altered functional and structural gradients, and tau distribution followed principal axes of functional and structural organization. Less distinct connectivity patterns, described by the authors as gradient contraction, were associated with greater tau burden. These findings support an association between reduced network differentiation, tau pathology, and cognition (Ottoy et al., 2024).

Preclinical amyloid studies provide a complementary perspective. He et al. studied cognitively unimpaired older adults with and without amyloid positivity and found substantially altered cortical hierarchies and reduced gradient variability in the amyloid-positive group. These hierarchy changes were related to automatically extractable spontaneous-speech features and to digit span performance, suggesting that gradient abnormalities may be detectable before objective clinical impairment and may connect early amyloid-related risk to subtle behavioral readouts (He et al., 2024).

Genetic risk may also modify gradient vulnerability. Song et al. examined DMN functional gradients in 271 participants stratified by diagnosis and APOE ε4 status. They found a group-by-genotype interaction in the right inferior temporal gyrus: AD APOE ε4 carriers showed the greatest gradient attenuation, whereas non-carriers showed a different, potentially compensatory pattern. In carriers, the right inferior temporal gradient correlated with several cognitive domains. Altered left superior frontal-to-right inferior temporal connectivity also mediated the association between amyloid burden and gradient disruption (Song et al., 2025). This is genotype-specific evidence that requires independent replication and does not yet support a clinical biomarker claim.

The entorhinal cortex provides a more anatomical route into the same question. Tan et al. integrated graph-theory-based stepwise connectivity with functional and structural connectome-gradient spaces to study entorhinal connectivity in AD. Because the entorhinal cortex is an early tau-vulnerable region, this approach helps translate local medial temporal vulnerability into multi-step network propagation along hierarchical axes (Tan et al., 2025). The finding should be read as a systems-level account of how early medial temporal pathology may affect distributed cortical hierarchy, rather than as a replacement for Braak staging or as a direct staging model (Braak and Braak, 1991).

### 4.4 Brain-behavior links: memory, global cognition, and subtle behavioral readouts

AD studies are most informative when they link gradient metrics to cognition or biology. Hu et al. (2022) linked regional gradient alterations to logical memory and dementia severity. He et al. (2023) associated reduced dispersion in aMCI and AD with cognitive scores. Wang et al. (2024) related gradient alterations to meta-analytic cognitive terms and gene-expression profiles, and Ottoy et al. (2024) linked gradient organization to tau and cognition in a multimodal cohort. Together, these studies show that direct gradient measures can carry behavioral and biological information beyond case-control separation.

The outcome measures remain heterogeneous, spanning global cognition, dementia severity, memory, speech features, and genetic-risk associations. Thus, the evidence supports behavioral relevance across analytic settings, not a clinically validated single-metric readout.

### 4.5 Boundary conditions and interpretation for the AD evidence

Direct AD studies report metric-specific changes in sensory-to-transmodal axis range, DMN-associated position, and network differentiation, with associations to cognition, amyloid, tau, APOE status, speech features, or gene-expression profiles. These findings support a bounded group-level inference rather than a clinical biomarker claim. As summarized in Table 3, the reported AD findings span whole-cortical functional-gradient analyses, gradient lateralization, DMN-focused analyses, multimodal functional–structural gradient studies, and related subcortical or network-topology extensions; these categories should not be interpreted as equivalent evidence for global principal-gradient compression.

Comparison across AD studies is limited by incomplete reporting of standardized effect sizes. Sample sizes range from small case-control cohorts to multi-scanner datasets, and most reports provide regional statistics, coefficients, correlations, or corrected maps rather than a uniform Cohen’s *d*. Supplementary Table 1 audits analytic scope, metrics, behavior/pathology associations, confound handling, and explicit incremental or external validation. No clearly negative whole-cortical AD gradient study comparable to the LBD functional-gradient null result was identified, but publication bias and analytic heterogeneity remain plausible.

Whether gradient abnormalities are specific to AD pathology, rather than a generic consequence of neurodegeneration, also requires comparison across disease spectra. In frontotemporal dementia, Bouzigues et al. (2025) reported metric-specific functional-gradient disruption that extended beyond the distribution of atrophy. This finding supports the general plausibility of disease-related hierarchy reorganization, but it does not establish a disease-nonspecific gradient phenotype or validate AD–LBD discrimination.

The field remains preclinical from a biomarker perspective. Many studies are cross-sectional, pipelines differ, and not all analyses test incremental value beyond atrophy, amyloid, tau, or conventional FC. Some studies examine cortical gradients, whereas others examine structural, subcortical, cerebellar, or thalamic gradients. For example, Yao et al. (2025) studied cerebello-thalamo-cortical (CTC) circuitry in ADNI-3 and reported CTC hyperconnectivity with thalamic and cerebellar gradient disruptions across AD, MCI, and control groups. Tan et al. (2025) examined stepwise entorhinal connectivity along connectomic gradients. This propagation/topology study is informative, but it does not provide direct evidence of whole-cortical principal-gradient compression. Such studies broaden the systems-neuroscience scope of AD but are not interchangeable with whole-cortical functional-gradient evidence.

**FIGURE 2:**
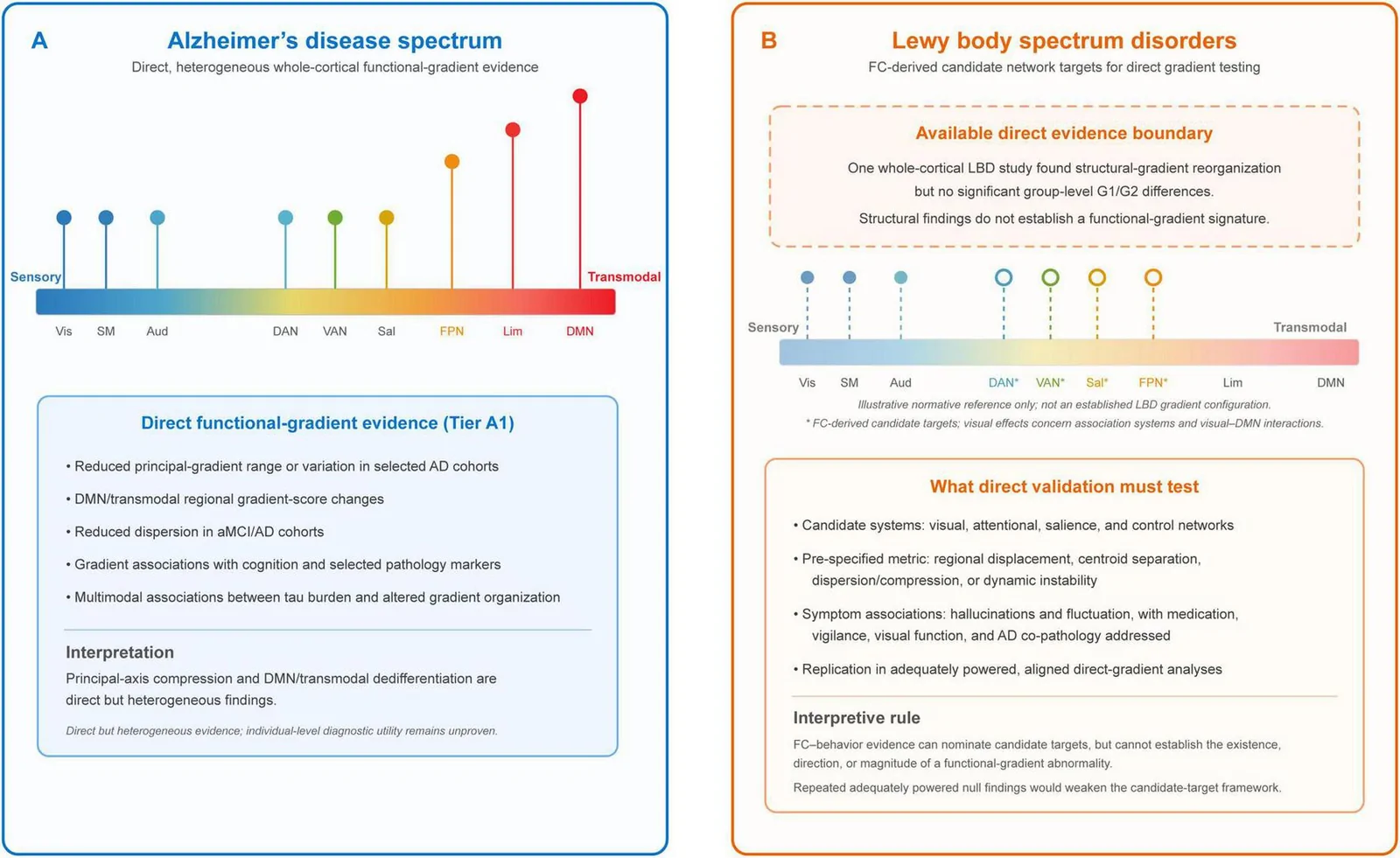
Evidence-weighted hierarchy-axis model for Alzheimer’s disease and Lewy body spectrum disorders. **(A)** The direct but heterogeneous whole-cortical functional-gradient evidence in Alzheimer’s disease (AD). Across studies, reported findings include metric-specific reduction in principal-gradient range or variation, default-mode network (DMN)/transmodal regional gradient-score changes, reduced dispersion in selected amnestic mild cognitive impairment and AD cohorts, and associations with cognition, pathology, and tau-related multimodal measures. These observations support direct but heterogeneous evidence for principal-gradient compression and DMN/transmodal dedifferentiation, while individual-level diagnostic utility remains unproven. **(B)** The more limited evidence in Lewy body spectrum disorders (LBD/PDD). The available whole-cortical study reported structural-gradient reorganization but no significant group-level differences in the first two static functional gradients (G1/G2); structural findings therefore do not establish a functional-gradient signature. Visual association, attentional, salience, and control systems are shown as FC-derived candidate network targets for direct gradient testing rather than as a validated LBD gradient configuration. Direct studies should prespecify the relevant gradient axis and metric, test symptom associations with hallucinations and cognitive fluctuation, address medication, vigilance, visual function, and mixed AD pathology, and include adequately powered replication. Asterisks indicate FC-derived candidate network targets, not validated gradient positions. FC–behavior evidence can nominate candidate targets but cannot establish the existence, direction, or magnitude of a functional-gradient abnormality.

Across the AD studies, convergence is strongest for altered principal-axis range or variation and reduced DMN–transmodal extremity, often with cognitive or molecular associations. Divergence concerns the affected component, exact metric, cohort definition, and analytic pipeline. AD therefore provides a direct but heterogeneous benchmark rather than a single canonical gradient phenotype. Figure 2 places this convergence and its limits alongside the much weaker LBD evidence base.

## 5 Lewy body spectrum disorders: limited direct gradient evidence and connectivity-derived candidate targets

### 5.1 Evidence audit: current state of direct gradient studies in DLB/PDD

The LBD-specific synthesis retained one core whole-cortical study with functional and structural gradient analyses, three adjacent gradient sources, and 11 indirect connectivity sources. Section 3.7, Supplementary Table 2, and Supplementary Figure 1 provide the search strategy, eligibility rules, and operational tiers. Consensus criteria defined DLB, prodromal LBD, PDD, and PD-MCI populations; contextual diagnostic literature did not contribute direct-gradient evidence (Emre et al., 2007; Litvan et al., 2012; Donaghy and McKeith, 2014; Gomperts, 2016; Jellinger and Korczyn, 2018; McKeith et al., 2017, 2020; Aarsland et al., 2021; Kucikova et al., 2024).

The core whole-cortical LBD-spectrum study is Zarkali et al. (2025), which examined 62 LBD participants, 46 PD patients with normal cognition, and 23 controls using structural and functional connectivity gradients. Its structural-gradient analyses constitute Tier A2 evidence: they identified widespread regional reorganization in LBD, and a composite structural-gradient difference score related to cognitive severity. Its functional-gradient analyses constitute Tier A1 evidence but were negative at the reported group level: the first two functional gradients showed no significant differences among LBD, PD-NC, and controls. Thus, the only current core LBD study supports preliminary structural-gradient reorganization but does not establish a broad static whole-cortical functional-gradient abnormality.

This negative Tier A1 result defines a current boundary: it specifies what cannot yet be claimed without proving that all functional-gradient effects are absent in LBD. It argues against an established broad static posterior attention-control signature but does not exclude symptom-stratified, focal, secondary-gradient, longitudinal, or time-resolved effects in larger cohorts. The current evidence therefore consists of preliminary Tier A2 structural findings and Tier C connectivity findings involving posterior visual, salience, and frontoparietal systems.

Li et al. (2025) reported striatal functional-gradient organization across Parkinson’s disease cognitive trajectories, including PDD. This supports the relevance of gradient approaches to Parkinsonian cognition but remains adjacent evidence: it concerns striatal rather than whole-cortical hierarchy gradients and does not include DLB.

Whole-cortical PD gradient studies are adjacent evidence, not direct DLB/PDD evidence. Wu et al. (2024) and Orlando et al. (2025) show that Parkinsonian neurobiology and dopamine state can alter cortical gradients. However, PD cohorts cannot be assumed to share the same pattern as DLB/PDD cohorts, which differ in dementia status, hallucinations, fluctuation, medication exposure, and diagnostic heterogeneity.

Conventional FC evidence is richer but remains indirect. It can nominate a direct-gradient target when the affected network has a known normative position, the FC pattern is directionally and spatially coherent, and the result is tied to a relevant phenotype. Candidate systems include visual, posterior cortical, attention, salience-control, thalamocortical, and frontoparietal networks. The required transition from FC to a direct gradient endpoint is specified in section 5.5.

### 5.2 Functional connectivity evidence for posterior attention-control disruption

Conventional FC studies provide the main empirical basis for the FC-derived candidate targets discussed here, but they require filtering. Lowther et al. (2014) reported reduced default-mode, salience, and executive connectivity in DLB, with greater basal-ganglia/limbic coupling and exploratory links to UPDRS and fluctuation scores. This supports broad DLB network vulnerability, but its spatial breadth is insufficient by itself to define a single visual-attention-control gradient axis. Structural network findings remain similarly indirect (Nicastro et al., 2021).

Schumacher et al. (2018) provided a complementary within- and between-network rs-fMRI analysis in 31 DLB patients, 29 AD patients, and 31 controls. Within-network connectivity was reduced in DLB mainly in motor, temporal, and frontal networks, whereas between-network connectivity was largely preserved and the DMN was relatively spared. The absence of significant symptom correlations limits direct behavioral interpretation, but the study helps define the conventional-FC background against which later hallucination- and fluctuation-focused findings should be judged.

Symptom- and stage-linked studies are more informative. Peraza et al. (2014) found relatively preserved DMN connectivity but reduced left frontoparietal, temporal, and sensorimotor connectivity in fluctuating DLB. Left frontoparietal desynchronization was related to fluctuation severity and frequency. Gabriel et al. (2025) reported reduced salience connectivity in DLB and lower salience/frontoparietal connectivity at the dementia stage. Salience connectivity was related to fluctuation, whereas frontoparietal connectivity was related to REM sleep behavior disorder and cognitive decline.

These findings nominate salience, attention, and frontoparietal systems as symptom-linked targets for direct testing because they occupy intermediate positions between sensory and transmodal extremes. Confidence is greatest when FC abnormalities are network-specific, symptom-linked, and replicated across cohorts; it is lower when results are diffuse, directionally inconsistent, or unrelated to core DLB features.

### 5.3 Hallucinations, aberrant sensory integration, and visual network instability

Visual hallucinations are the clearest behavioral test case because multiple studies link them to visual-attention interactions rather than isolated visual cortex dysfunction. Mehraram et al. (2022) found weakened visual ventral connectivity and reduced coupling with default-mode and ventral attentional networks in hallucinating Lewy body dementia, with structural links implicating nucleus basalis of Meynert and thalamic pathways.

Firbank et al. provided complementary rs-fMRI evidence in a larger Lewy body disease cohort: 41 participants with visual hallucinations, 48 without hallucinations, and 60 controls. They tested connectivity between visual cortex, ventral attention, dorsal attention, and default-mode regions. Participants with hallucinations showed reduced connectivity within the ventral attention network and between visual and default-mode networks. Visual-DMN connectivity correlated with pareidolia task performance, whereas ventral attention connectivity correlated with visuospatial performance (Firbank et al., 2024).

Earlier hallucination-focused DLB studies provide compatible but still indirect evidence. Pezzoli et al. (2021) reported altered thalamic, parietal, putaminal, visual, and DMN connectivity in hallucinating DLB. Zorzi et al. (2021) used FDG-PET/MR metabolic connectivity and found visual, dorsal-attention, DMN, insular, and orbitofrontal alterations associated with hallucinations. These studies support cross-modal plausibility but do not demonstrate cortical-gradient displacement or compression.

Overall, hallucination-focused FC, EEG, and metabolic-connectivity studies nominate visual-attention-DMN systems as high-priority targets for direct gradient testing. They provide symptom-relevant Tier C evidence, but none can establish the existence, direction, magnitude, or metric of a functional-gradient abnormality. A future direct analysis must pre-specify whether it tests regional displacement, between-network separation, axis compression, or time-resolved instability.

### 5.4 Cognitive fluctuation and salience-control dysfunction

Gabriel et al. (2025) provide the strongest stage- and symptom-linked FC evidence for a salience-control candidate target. They reported reduced salience connectivity in DLB, additional frontoparietal impairment at the dementia stage, and associations with fluctuation, REM sleep behavior disorder, and cognitive decline.

These findings motivate a direct test of whether cognitive fluctuation relates to pre-specified regional displacement or time-resolved instability of salience and frontoparietal systems. Static displacement and temporal instability should be tested separately because fluctuation measures and implicated control/arousal systems vary across studies.

Static gradients may be poorly matched to fluctuation. Dynamic FC studies suggest time-varying network states in DLB, but current data do not establish dynamic gradient biomarkers (Sourty et al., 2016; Schumacher et al., 2021). Future work may therefore need time-resolved gradient or state-space approaches when fluctuation is the target phenotype.

### 5.5 Methodological bridge: when can FC evidence nominate direct-gradient targets?

The transition from conventional FC findings to direct-gradient targets must be explicit and one-directional. FC can nominate a candidate gradient target but cannot by itself establish the direction, magnitude, metric, or presence of a gradient abnormality. Three requirements guide target nomination. First, the affected network must have a well-characterized normative position in gradient space. Second, the FC pattern must be sufficiently coherent in direction and spatial distribution to motivate a specific direct test rather than merely indicate diffuse disorganization. Third, the planned direct endpoint must be specified in advance: global compression, regional displacement, between-network boundary blurring, a secondary-axis effect, or time-resolved instability. These criteria are informed by concerns about gradient estimation and alignment (Vos de Wael et al., 2020), spatial-autocorrelation bias (Burt et al., 2020), and overinterpretation of connectopic or gradient-like maps (Watson and Andrews, 2023). Replication and symptom linkage modify confidence but do not independently establish a gradient effect. The operational FC-to-gradient candidate-target filter is summarized in Table 5.

**TABLE 5:** FC-to-gradient candidate-target filter for Lewy body spectrum disorders.

Candidate direct-gradient target	Motivating evidence	Core eligibility criteria	Confidence modifiers	Current status	Action in manuscript
Visual–attention–control systems as candidate direct-gradient targets	Visual association, attention, frontoparietal, salience, and DMN FC findings (Lowther et al., 2014; Peraza et al., 2014; Firbank et al., 2024; Gabriel et al., 2025).	Network specificity is plausible; direct endpoint, gradient direction, and axis identity are not established.	Replication incomplete; symptom links indirect or variable.	FC-derived candidate target; not an established gradient target.	Retain only as a dashed target for direct testing; do not depict an established arc or axis.
Hallucination severity maps to a pre-specified visual–attention gradient metric	Hallucination-focused FC/structural studies (Mehraram et al., 2022; Firbank et al., 2024).	Visual and attention network specificity is strong, but direct metric and direction remain unknown.	Symptom link relatively strong at FC level; replication still required.	High-priority direct test; not direct evidence.	Retain as a dashed test target in Figure 3; specify the metric in future studies.
Salience/control spatial displacement or temporal instability predicts cognitive fluctuation	Fluctuation- and disease-stage-related frontoparietal/salience findings (Peraza et al., 2014; Gabriel et al., 2025).	Network specificity plausible; static versus dynamic endpoint and direction remain uncertain.	Symptom link plausible but measurement of fluctuation varies across studies.	Speculative direct-test target.	Mention cautiously; require pre-specified static or time-resolved metric.
General LBD functional-gradient compression	Sparse direct gradient evidence; core functional-gradient study negative.	Not satisfied.	No robust replication or symptom–gradient link.	Do not present as established.	Exclude from main comparative claim.

FC, functional connectivity; LBD, Lewy body disease; VAN, ventral attention network; DMN, default-mode network. Core eligibility requirements for target nomination (all three must be met): (1) network specificity: the affected network has a well-characterized position in normative gradient space; (2) directional and spatial coherence: the FC pattern motivates a focused direct test rather than diffuse global disorganization; and (3) metric specification: the proposed direct outcome is stated in advance as global compression, regional displacement, between-network boundary blurring, a secondary-axis effect, or time-resolved instability. Replication and symptom linkage modify confidence but are not proof of a gradient effect. A conventional FC study can nominate a candidate target; it cannot establish the existence, direction, or magnitude of a functional-gradient abnormality.

FC findings do not justify target nomination when they are directionally inconsistent, involve networks with uncertain normative coordinates, span multiple axes without specificity, arise from a single small unreplicated study, or lack a relevant phenotype. A candidate target should be downgraded or rejected when an adequately powered, preregistered analysis of aligned gradients fails to detect the specified axis or symptom association across appropriate sensitivity analyses. The same applies when an apparent effect is no more specific than diffuse global change.

### 5.6 From connectivity abnormalities to testable hierarchy-axis models

After filtering, three LBD/PDD questions remain: whether visual, attentional, salience, and control systems map onto a reproducible secondary-gradient target; whether hallucination severity relates to a pre-specified direct gradient metric; and whether cognitive fluctuation relates to static displacement or time-resolved instability of salience/thalamocortical-control systems. None has been validated by direct functional-gradient studies.

The LBD section therefore yields a focused research agenda. Current evidence does not establish functional-gradient disruption, but it identifies network- and symptom-specific targets that can be tested using standardized, preregistered gradient pipelines.

## 6 An asymmetric comparative framework

Section 6 operationalizes the evidence asymmetry rather than restating it. Its purpose is to define what can be compared across disease spectra and what remains untested. Table 1 integrates the cross-disease comparison by distinguishing established AD findings, LBD/PDD FC-derived candidate targets, and unresolved mixed-pathology and validation questions.

### 6.1 Evidence-weighted comparison

AD has direct but heterogeneous whole-cortical findings, including metric-specific principal-gradient or DMN-transmodal changes with cognitive associations. In the LBD/PDD audit, direct structural evidence is preliminary and the only core static functional study was negative at group level. The comparison therefore uses direct metrics for AD and explicitly labeled FC-derived targets for LBD/PDD, as reflected in Tables 3–5 and the validation agenda.

### 6.2 Shared principle: loss of hierarchical differentiation

The phrase “shared principle” is an epistemic scaffold rather than a proven cross-disease mechanism. In AD, direct studies support metric-specific sensory-to-transmodal changes and reduced DMN-transmodal extremity. In LBD, posterior cortical and attention-control FC abnormalities nominate a possible secondary-gradient target; repeated negative direct studies would require revision of the hierarchy-axis framework.

### 6.3 Divergent candidate hierarchy axes

The comparative proposal is a differential test design, not a claim of two established disease axes. It asks whether direct analyses continue to support AD-associated changes along the principal sensory-to-transmodal/DMN axis and whether LBD/PDD shows a directly measured effect in a secondary visual-attention-control configuration. The LBD/PDD component remains an FC-derived, symptom-linked candidate target with no specified direction or magnitude.

### 6.4 Mixed AD-Lewy pathology as a stress test of the hierarchy framework

Mixed AD–Lewy pathology should be operationalized rather than inferred from syndromic overlap alone. A pragmatic *in-vivo* scheme should define the AD component with amyloid and tau biomarkers and the Lewy body component with a clinically probable DLB/PDD phenotype plus dopamine-transporter imaging where available. One stringent three-stratum design would define AD-predominant cases as amyloid-positive and tau-positive without convincing clinical or dopaminergic support for Lewy body disease. Lewy body-predominant cases would have clinically probable DLB/PDD, an abnormal dopamine-transporter study, and absent or low AD biomarker burden. Mixed AD–Lewy cases would have clinically probable DLB/PDD, dopaminergic support, and concurrent amyloid and tau positivity. Dopamine-transporter imaging supports nigrostriatal degeneration but is not stand-alone pathological confirmation of Lewy body disease. Ante-mortem imaging linked to autopsy remains the reference standard for validating these *in-vivo* strata (Jack et al., 2018; McKeith et al., 2017).

These pre-specified strata offer a falsifiable test of the hierarchy framework. If AD and LBD affect largely independent axes, mixed AD–Lewy cases should show additive abnormalities: reduced principal-gradient range associated with the AD biomarker component and a directly measured secondary visual-attention-control effect associated with the Lewy body component. If the disorders converge on a shared vulnerability axis, mixed cases should show greater severity on a common gradient dimension. A third possibility is subadditive or compensatory reorganization, in which coexisting pathologies, medication exposure, or adaptive network responses reduce, mask, or relocate effects. Gradient profiles should therefore be analyzed against continuous amyloid, tau, dopaminergic, and clinical measures as well as categorical strata, with adjustment for dementia severity and atrophy.

### 6.5 Diagnostic specificity and current validation status

Validation status should be defined by observable milestones rather than subjective readiness labels. A research-stage finding is limited to an aligned group-level association. A candidate biomarker additionally requires repeatable individual-level discrimination, external validation, and incremental performance beyond conventional FC, structural MRI, and established molecular or dopaminergic markers. Clinical evaluation further requires test–retest and site/scanner robustness, calibrated predictions, and demonstrable utility within a pre-specified decision context.

Neither disease spectrum currently meets these milestones in full. AD has direct but heterogeneous group-level evidence but lacks a replicated individual-level gradient-based classifier, an external validation program, and a clinical-utility analysis. LBD lacks replicated direct whole-cortical functional-gradient evidence and includes an important group-level null result. AD and LBD gradient metrics therefore remain research-stage measures rather than clinically deployable diagnostic biomarkers.

### 6.6 Evidence-status summary: WHAT can and cannot be claimed now

To avoid conflating direct evidence, FC-derived candidate targets, and clinical translation, the current evidence permits only the tier-specific conclusions summarized in Table 6. AD has emerging direct but heterogeneous whole-cortical functional-gradient evidence. For LBD/PDD, current structural-gradient and FC findings define candidate targets for direct testing rather than an established functional-gradient signature.

**TABLE 6:** Evidence-status summary: permissible conclusions and current limits.

Evidence status	Permissible conclusion
Emerging direct evidence	AD: direct but heterogeneous whole-cortical functional-gradient studies support metric-specific principal-gradient / DMN-transmodal disruption with cognition/pathology associations.
Preliminary direct and indirect evidence	LBD: preliminary Tier A2 structural-gradient reorganization. Tier C FC-behavior findings nominate visual, attention, salience, and control systems for direct testing.
Not yet established	A replicated LBD/PDD functional-gradient signature; a validated visual-attention-control axis; the direction or magnitude of an LBD gradient effect; or a dynamic gradient biomarker.
Not supportable for clinical use	Individual-level diagnosis, prognosis, or management decisions based on gradient metrics in AD or LBD.

AD, Alzheimer’s disease; DMN, default-mode network; FC, functional connectivity; LBD, Lewy body disease; PDD, Parkinson’s disease dementia. Tier A2 denotes direct whole-cortical structural-gradient evidence, whereas Tier C denotes indirect FC/connectivity evidence. Tier A2 structural-gradient findings do not establish a whole-cortical functional-gradient signature, and Tier C findings can nominate candidate targets for direct testing but cannot establish a functional-gradient effect.

## 7 Cross-disease brain-behavior mapping: a comparative synthesis

This section asks whether comparable cognitive or behavioral dimensions map onto different hierarchy axes across AD and Lewy body spectrum disorders. The phenotype-level comparison is organized according to the evidence-weighted matrix in Table 1; the table distinguishes direct AD gradient–behavior evidence from LBD/PDD network findings that currently identify candidate targets rather than validated gradient phenotypes.

### 7.1 Episodic memory decline: DMN-transmodal disruption in AD versus relative preservation in early LBD

Episodic memory decline is a defining early feature of typical AD and is anatomically linked to medial temporal, posterior cingulate, precuneus, and angular gyrus systems that occupy the transmodal end of the cortical hierarchy. The gradient framework reframes this clinical pattern as a testable failure of DMN-transmodal differentiation: memory impairment may reflect both medial temporal damage and reduced separation between integrative transmodal systems and lower-order networks (Hu et al., 2022; Jack et al., 2018).

In early DLB, memory impairment is often less prominent than visuospatial and attentional deficits, although mixed AD pathology can substantially modify this profile. This contrast provides a behavioral benchmark for the hierarchy-axis framework. If AD preferentially affects the principal sensory-to-transmodal axis and LBD preferentially affects visual-attention-control systems, memory-dominant phenotypes should relate more strongly to principal-gradient abnormalities than to the candidate LBD secondary axis. Direct comparative studies are needed to test this prediction.

### 7.2 Visual hallucinations: uncommon in typical AD but prevalent in DLB: a gradient-axis signature?

Persistent formed visual hallucinations are uncommon in typical early AD but are a core clinical feature of DLB, especially when they occur early or repeatedly (McKeith et al., 2017). Available neuroimaging findings motivate direct tests of visual-attention and salience-control systems rather than a prespecified hierarchy abnormality. Hallucinations can also occur in advanced AD, mixed AD-Lewy pathology, medication effects, or delirium.

The candidate prediction is that hallucinations reflect altered coupling between visual association regions and attention-control systems rather than dysfunction of the visual network alone. A direct study should pre-specify an aligned outcome, such as regional displacement, between-network separation, global axis range, or time-resolved instability. It should also adjust for global cognition, visual acuity, dopaminergic medication exposure, and AD co-pathology.

### 7.3 Cognitive fluctuation: a Lewy body-specific behavioral signature

Cognitive fluctuation is a distinctive clinical feature of DLB and a useful target for testing whether LBD alters cortical organization differently from AD (McKeith et al., 2017). Conventional connectivity studies implicate thalamocortical loops, salience-network instability, and attention-control interactions. The relevant direct-gradient question is not a presumed principal-axis compression. It is whether intermediate control systems show static regional displacement or time-resolved instability while regulating transitions between sensory processing and higher-order cognition (Gabriel et al., 2025; Peraza et al., 2014).

A rigorous test would require repeated or dynamic resting-state acquisitions, fluctuation-sensitive clinical measures, and gradient metrics that capture temporal variability as well as spatial position. Cognitive fluctuation therefore remains an FC-derived target for gradient validation rather than an established gradient-behavior association.

### 7.4 Executive impairment: shared phenotype, potentially distinct hierarchy mechanisms

Executive dysfunction occurs in both AD and LBD, making it useful for separating behavioral similarity from mechanistic similarity. In AD, executive deficits may arise as frontoparietal systems become disconnected from an altered transmodal hierarchy. In LBD, they may reflect degraded attention-control coupling, salience-network instability, and fluctuating thalamocortical engagement. Similar behavioral endpoints may therefore arise from different hierarchy-level disturbances (Gabriel et al., 2025; Hu et al., 2022; McKeith et al., 2017).

This distinction matters clinically. Neuropsychological tests alone may identify executive impairment without distinguishing AD-related transmodal change, LBD-related attentional-control instability, or mixed pathology. Gradient imaging could in principle provide a systems-level phenotype that separates these mechanisms, but this remains a research question requiring head-to-head AD, DLB, PDD, and mixed-pathology cohorts.

### 7.5 Summary: comparative mapping across the 2 × N matrix

**FIGURE 3:**
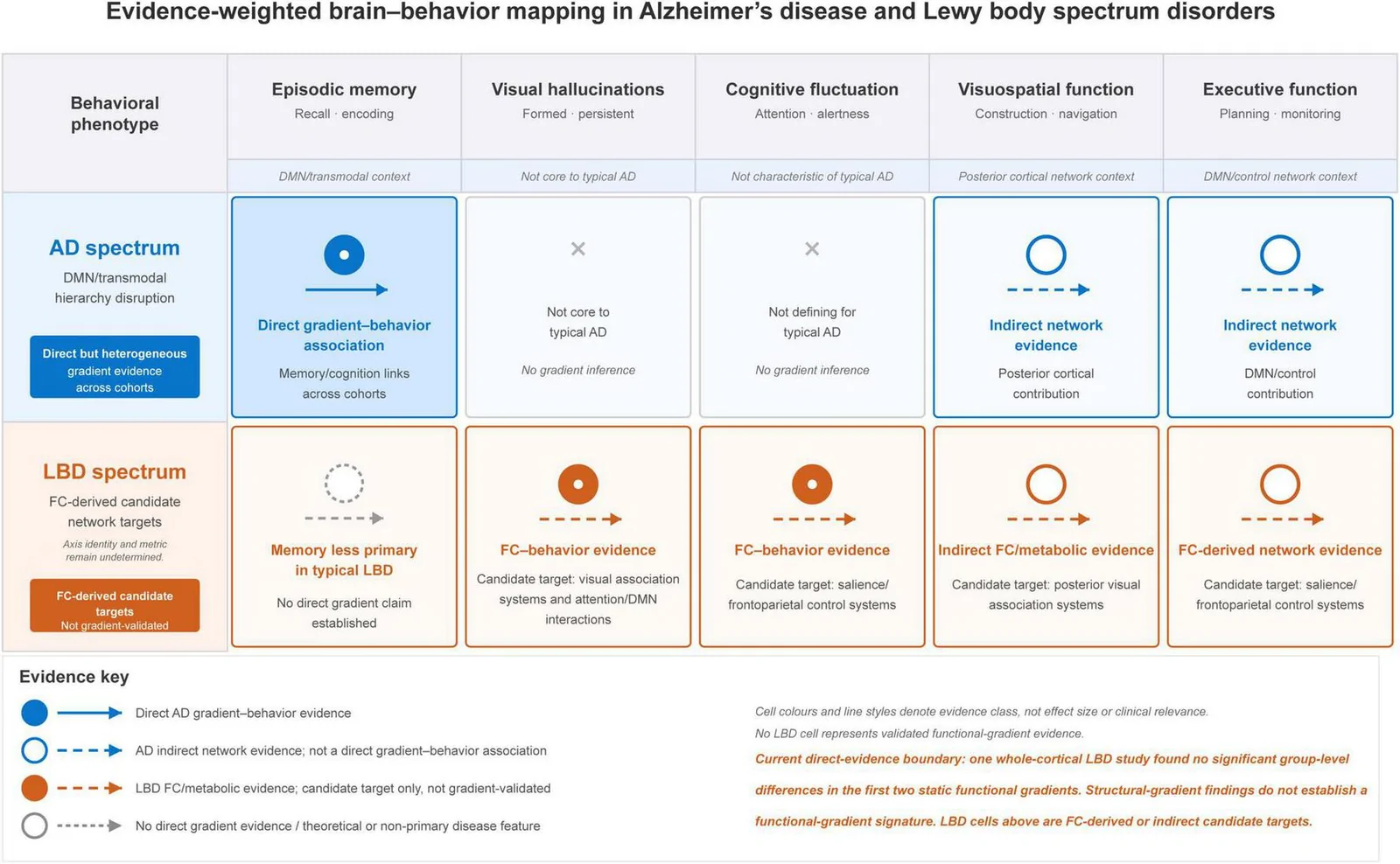
Evidence-weighted brain–behavior mapping across Alzheimer’s disease and Lewy body spectra. The figure summarizes the evidentiary status of proposed brain–behavior relationships across episodic memory, visual hallucinations, cognitive fluctuation, visuospatial function, and executive function. In Alzheimer’s disease (AD), episodic memory shows direct gradient–behavior evidence involving default-mode network (DMN)/transmodal organization, whereas visuospatial and executive deficits are supported mainly by indirect network-level evidence. Visual hallucinations and cognitive fluctuation are not defining features of typical AD and do not support gradient inference. In Lewy body spectrum disorders (LBD/PDD), visual hallucinations, cognitive fluctuation, visuospatial dysfunction, and executive impairment are supported primarily by functional-connectivity, metabolic-connectivity, or network-level findings that identify candidate targets involving visual association, attentional, salience, and frontoparietal control systems. These findings are not gradient-validated. The available whole-cortical LBD study found no significant group-level differences in the first two static functional gradients; structural-gradient findings therefore do not establish a functional-gradient signature. Colors and line styles indicate evidence class rather than effect size or clinical relevance.

The 2 × N matrix is a structured research agenda rather than a completed taxonomy (Figure 3). It maps which brain-behavior cells are supported by direct gradient evidence, which are inferred from FC-behavior findings, and which remain direct-testing targets.

## 8 Clinical and translational implications

### 8.1 Gradient-informed dementia phenotyping

Conventional dementia phenotyping is limited because neuropsychological profiles and regional imaging abnormalities do not always map cleanly onto underlying pathology. Functional gradients may help bridge this gap by representing cognition within a macroscale hierarchy rather than as the output of isolated networks. In AD, directly measured reductions in principal-gradient range provide a plausible systems-level marker of transmodal network dedifferentiation. Such measures may complement hippocampal atrophy by capturing distributed loss of cortical differentiation that is not reducible to focal tissue loss.

For LBD, phenotyping remains a research target. The framework identifies posterior visual, attention, salience, and control systems for pre-specified direct gradient testing in patients with hallucinations, cognitive fluctuation, or visuospatial impairment. It does not imply a known axis, direction, or metric of LBD/PDD functional-gradient change.

### 8.2 Differential diagnosis potential and current limitations

The comparative framework suggests a diagnostic question rather than a validated classifier: whether AD-associated principal-axis changes and a directly measured LBD posterior attention-control effect provide complementary individual-level information. Translation would require the milestones specified in Section 6.5, including external validation, reliability across sites and acquisitions, calibrated prediction, incremental value beyond established biomarkers, and decision-analytic net benefit.

Neither disease spectrum currently meets the requirements for a clinically usable gradient biomarker. Gradient analysis should therefore be framed as a research route to differential network phenotypes, not as a diagnostic test.

If validated, the most plausible clinical use would not be routine screening of typical AD or typical DLB. More realistic scenarios include atypical early onset dementia, discordant clinical and molecular profiles, suspected mixed AD-Lewy pathology, and patients in whom hallucinations, visuospatial dysfunction, or fluctuation dominate despite equivocal standard imaging. Even in these settings, gradient profiling would have to be interpreted as an adjunct to clinical examination, neuropsychology, molecular biomarkers, and structural imaging rather than as a stand-alone diagnostic test.

### 8.3 Integration with PET, structural MRI, and multimodal biomarkers

Gradient metrics should be considered complementary to established biomarker systems rather than replacements. Amyloid and tau PET identify molecular substrates of AD, whereas structural MRI characterizes atrophy and regional vulnerability. Dopamine-transporter imaging and related markers help identify nigrostriatal degeneration in Lewy body disorders. Functional gradients may capture a different layer: the cortical network consequences of pathology. This layered model is more realistic than a competing-biomarker narrative.

A useful multimodal model would ask whether molecular burden predicts directly measured gradient changes, whether those changes mediate the relationship between pathology and cognition, and whether the mediation differs between AD and LBD. In AD, tau burden in transmodal cortices may align with reduced principal-gradient range and memory decline. In LBD, dopaminergic or cholinergic vulnerability may interact with visual and attentional network instability, but the relevant direct-gradient signature remains to be established. These are mechanistic questions, not settled pathways.

### 8.4 Minimum study design and validation standards

A minimum validation protocol should justify sample size by a pre-specified effect-size or performance target, expected attrition from motion and quality control, and the need for independent validation. It should recruit biomarker-supported or pathology-confirmed AD, LBD, and mixed-pathology groups; use sufficiently long 3T resting-state fMRI with vigilance monitoring; report relevant medication exposure; harmonize preprocessing and motion control; pre-specify parcellation, affinity construction, embedding, alignment, and exact gradient outcomes; and include a test–retest subset where feasible. The central analysis should test whether those metrics explain hallucinations or fluctuation beyond conventional FC, atrophy, molecular biomarkers, and clinical variables.

Machine-learning analyses are informative only when they test incremental value rather than merely add model complexity. Models should compare conventional FC alone, gradient metrics alone, and combined FC-gradient features using nested cross-validation and a locked external validation set. Reporting should include discrimination, calibration, and decision-analytic net benefit, rather than only an internally optimized area under the curve. The central question is whether gradient-axis metrics improve prediction of cognition, hallucinations, fluctuation, or diagnostic group beyond conventional FC and established biomarkers.

Where evidence is limited, machine learning should remain a future validation strategy rather than an established translational application.

## 9 Future directions and testable predictions

The following agenda derives from the specific gaps identified in Sections 5–8. Each direction is framed as a testable prediction or validation requirement for the hierarchy-axis framework. The unresolved cells in Table 1 define the principal validation agenda: whether LBD/PDD has a reproducible whole-cortical functional-gradient signature, how mixed AD–Lewy pathology modifies hierarchy-level organization, and whether gradient metrics add clinically meaningful information beyond conventional FC and established biomarkers.

### 9.1 Does LBD exhibit a reproducible secondary-gradient effect in posterior attention-control systems?

The first priority is to determine whether DLB and PDD show a reproducible, directly measured secondary-gradient effect in posterior attention-control systems. A decisive study would include pathology-confirmed or biomarker-supported DLB/PDD, AD, and healthy controls; use harmonized resting-state fMRI; align individual gradients to a common template; and test pre-specified metrics in visual association, attention, salience, and frontoparietal systems. Reporting should include acquisition duration, motion thresholds, parcellation, alignment, anatomical component labels, and behavioral tests. For cognitive fluctuation, repeated or time-resolved acquisitions may be more informative than a single static estimate (Allen et al., 2014; Preti et al., 2017; Brown et al., 2022; Lurie et al., 2020).

A positive result would support the candidate LBD model. A repeated null result would be equally informative: if well-powered studies continue to find conventional FC abnormalities but no corresponding directly estimated gradient effect, the framework would require revision, because static gradient geometry may not capture the relevant network instability.

### 9.2 Do mixed AD-LBD cases show additive or dominant-axis hierarchy abnormalities?

Section 6.4 specifies the biomarker strata required for mixed-pathology analyses. Future studies should fix those strata before gradient analysis and retain continuous amyloid, tau, and dopaminergic measures for sensitivity analyses. They should then test whether mixed cases show additive principal- and secondary-gradient effects, greater severity on a shared dimension, or subadditive/compensatory reorganization. This requires ante-mortem imaging linked to autopsy or a high-confidence, pre-specified multimodal biomarker classification.

This prediction makes the framework falsifiable by specifying how overlapping pathologies could produce additive, dominant-axis, or interactive effects.

### 9.3 Do gradient metrics add information beyond conventional FC?

The strongest methodological test is incremental validity. Future studies should determine whether gradient metrics improve prediction of cognitive-behavioral phenotypes beyond conventional FC, structural MRI, molecular biomarkers, and clinical variables. The relevant standard is improved external discrimination, calibration, interpretability, and decision-analytic net benefit, not statistical significance alone.

This test is particularly important for LBD. If visual and attentional FC abnormalities predict hallucinations but gradient metrics do not improve prediction, the gradient framework may be conceptually useful but clinically redundant. Conversely, improved prediction of hallucinations or cognitive fluctuation would indicate that hierarchy-axis geometry captures information not represented in conventional FC summaries.

### 9.4 Do gradient abnormalities precede symptom emergence?

Cross-sectional studies cannot determine whether directly measured gradient changes are early mechanisms, downstream consequences, or compensatory reorganization. Longitudinal studies in prodromal cohorts are therefore essential. For AD, relevant cohorts include subjective cognitive decline and MCI with amyloid and tau characterization. For LBD, isolated REM sleep behavior disorder offers a potential prodromal population (Postuma et al., 2019), although phenoconversion pathways are heterogeneous. If a candidate posterior attention-control effect is present only at the dementia stage, RBD cohorts would show no abnormality. This informative null would narrow the disease-trajectory window for gradient emergence.

The key test is temporal ordering. If a directly measured reduction in principal-gradient range precedes cognitive decline, it may serve as an early systems marker. If it appears only after symptoms emerge, it may be better interpreted as a state marker of established network dysfunction. If an effect tracks cognitive fluctuation, dynamic or time-resolved gradient methods may be required instead of static resting-state embeddings.

### 9.5 Toward pathology-informed, hierarchy-guided dementia classification

The long-term goal is a hierarchy-informed layer of dementia classification that distinguishes principal transmodal, posterior attention-control, salience-control, and mixed patterns. Clinical adoption would require pathological calibration, test–retest and multi-site reliability, external validation, and clinically meaningful incremental value within established AD and Lewy body biomarker frameworks (Jack et al., 2013, 2018; Knopman et al., 2021; Scheltens et al., 2021; McKeith et al., 2017).

Until these conditions are met, gradient analysis should be presented as an organizing framework and research tool rather than a deployable clinical assay. This position is conservative but justified: the framework identifies the evidence required before clinical translation can be supported.

## 10 Conclusion

This review presents an evidence-weighted hierarchy-axis framework for AD and Lewy body spectrum disorders. In AD, direct but heterogeneous studies support metric-specific changes at the principal-gradient/DMN-transmodal end of cortical organization, with cognitive associations. These findings remain insufficient for clinical use because longitudinal replication and incremental value beyond established measures have not yet been demonstrated.

For LBD/PDD, direct evidence does not establish a static whole-cortical functional-gradient signature. Visual-attention, salience, and control-network findings are therefore FC-derived targets for direct tests, not known gradient abnormalities.

The framework separates direct observations from indirect target nomination and specifies the evidence needed for clinical translation. Whether current gradient measures explain cognitive or behavioral variance beyond conventional FC remains unresolved, because direct incremental comparisons are rare and have not been synthesized quantitatively. The framework now guides reproducible, metric-specific tests of LBD/PDD effects, mixed pathology, and incremental value beyond conventional connectivity and established biomarkers.

## References

[B1] Aarsland D. Batzu L. Halliday G. Geurtsen G. Ballard C. Ray Chaudhuri K. et al.. (2021). Parkinson disease-associated cognitive impairment. *Nat. Rev. Dis. Primers* 7:47. 10.1038/s41572-021-00280-3 34210995

[B2] Albert M. DeKosky S. Dickson D. Dubois B. Feldman H. Fox N. et al.. (2011). The diagnosis of mild cognitive impairment due to Alzheimer’s disease: Recommendations from the National Institute on Aging-Alzheimer’s Association workgroups on diagnostic guidelines for Alzheimer’s disease. *Alzheimers Dement.* 7 270–279. 10.1016/j.jalz.2011.03.008 21514249 PMC3312027

[B3] Allen E. Damaraju E. Plis S. Erhardt E. Eichele T. Calhoun V. (2014). Tracking whole-brain connectivity dynamics in the resting state. *Cereb. Cortex* 24 663–676. 10.1093/cercor/bhs352 23146964 PMC3920766

[B4] Bernhardt B. Smallwood J. Keilholz S. Margulies D. (2022). Gradients in brain organization. *Neuroimage* 251:118987. 10.1016/j.neuroimage.2022.118987 35151850

[B5] Bethlehem R. Paquola C. Seidlitz J. Ronan L. Bernhardt B. Consortium C. et al.. (2020). Dispersion of functional gradients across the adult lifespan. *Neuroimage* 222:117299. 10.1016/j.neuroimage.2020.117299 32828920 PMC7779368

[B6] Bouzigues A. Godefroy V. Du V. Russell L. L. Houot M. Le Ber I. et al.. (2025). Disruption of macroscale functional network organisation in patients with frontotemporal dementia. *Mol. Psychiatry* 30 2436–2447. 10.1038/s41380-024-02847-4 39580607 PMC12092252

[B7] Braak H. Braak E. (1991). Neuropathological stageing of Alzheimer-related changes. *Acta Neuropathol.* 82 239–259. 10.1007/BF00308809 1759558

[B8] Brown J. Lee A. Pasquini L. Seeley W. W. (2022). A dynamic gradient architecture generates brain activity states. *Neuroimage* 261:119526. 10.1016/j.neuroimage.2022.119526 35914669 PMC9585924

[B9] Buckner R. DiNicola L. (2019). The brain’s default network: Updated anatomy, physiology and evolving insights. *Nat. Rev. Neurosci.* 20 593–608. 10.1038/s41583-019-0212-7 31492945

[B10] Burt J. Demirtaş M. Eckner W. Navejar N. Ji J. Martin W. et al.. (2018). Hierarchy of transcriptomic specialization across human cortex captured by structural neuroimaging topography. *Nat. Neurosci.* 21 1251–1259. 10.1038/s41593-018-0195-0 30082915 PMC6119093

[B11] Burt J. Helmer M. Shinn M. Anticevic A. Murray J. (2020). Generative modeling of brain maps with spatial autocorrelation. *Neuroimage* 220:117038. 10.1016/j.neuroimage.2020.117038 32585343

[B12] Ciric R. Wolf D. Power J. Roalf D. Baum G. Ruparel K. et al.. (2017). Benchmarking of participant-level confound regression strategies for the control of motion artifact in studies of functional connectivity. *Neuroimage* 154 174–187. 10.1016/j.neuroimage.2017.03.020 28302591 PMC5483393

[B13] Coifman R. Lafon S. (2006). Diffusion maps. *Appl. Comput. Harmonic Anal.* 21 5–30. 10.1016/j.acha.2006.04.006

[B14] Donaghy P. McKeith I. (2014). The clinical characteristics of dementia with Lewy bodies and a consideration of prodromal diagnosis. *Alzheimers Res. Ther.* 6:46. 10.1186/alzrt274 25484925 PMC4255387

[B15] Dubois B. Feldman H. Jacova C. Hampel H. Molinuevo J. Blennow K. et al.. (2014). Advancing research diagnostic criteria for Alzheimer’s disease: The IWG-2 criteria. *Lancet Neurol.* 13 614–629. 10.1016/S1474-4422(14)70090-0 24849862

[B16] Emre M. Aarsland D. Brown R. (2007). Clinical diagnostic criteria for dementia associated with Parkinson’s disease. *Mov. Disord.* 22 1689–1707. 10.1002/mds.21507 17542011

[B17] Esteban O. Markiewicz C. Blair R. Moodie C. Isik A. Erramuzpe A. et al.. (2019). fMRIPrep: A robust preprocessing pipeline for functional MRI. *Nat. Methods* 16 111–116. 10.1038/s41592-018-0235-4 30532080 PMC6319393

[B18] Firbank M. Collerton D. Morgan K. Schumacher J. Donaghy P. O’Brien J. et al.. (2024). Functional connectivity in Lewy body disease with visual hallucinations. *Eur. J. Neurol.* 31:e16115. 10.1111/ene.16115 37909801 PMC11235993

[B19] Fischl B. (2012). FreeSurfer. *Neuroimage* 62 774–781. 10.1016/j.neuroimage.2012.01.021 22248573 PMC3685476

[B20] Gabriel V. Chabran E. Sourty M. Cretin B. Philippi N. Muller C. et al.. (2025). Salience and frontoparietal network impairments across disease stages in dementia with Lewy bodies: A comparative functional MRI study with Alzheimer’s disease. *J. Alzheimers Dis.* 108 1398–1409. 10.1177/13872877251386844 41105629

[B21] Gao Z. Zheng L. Krieger-Redwood K. Halai A. Margulies D. Smallwood J. et al.. (2022). Flexing the principal gradient of the cerebral cortex to suit changing semantic task demands. *Elife* 11:e80368. 10.7554/eLife.80368 36169281 PMC9555860

[B22] Glasser M. Coalson T. Robinson E. Hacker C. Harwell J. Yacoub E. et al.. (2016). A multi-modal parcellation of human cerebral cortex. *Nature* 536 171–178. 10.1038/nature18933 27437579 PMC4990127

[B23] Gomperts S. (2016). Lewy body dementias: Dementia with lewy bodies and parkinson disease dementia. *Continuum* 22 435–463. 10.1212/CON.0000000000000309 27042903 PMC5390937

[B24] He R. Al-Tamimi J. Sánchez-Benavides G. Montaña-Valverde G. Domingo Gispert J. Grau-Rivera O. et al.. (2024). Atypical cortical hierarchy in Aβ-positive older adults and its reflection in spontaneous speech. *Brain Res.* 1830:148806. 10.1016/j.brainres.2024.148806 38365129

[B25] He Y. Li Q. Fu Z. Zeng D. Han Y. Li S. (2023). Functional gradients reveal altered functional segregation in patients with amnestic mild cognitive impairment and Alzheimer’s disease. *Cereb. Cortex* 33 10836–10847. 10.1093/cercor/bhad328 37718155

[B26] Hu Q. Li Y. Wu Y. Lin X. Zhao X. (2022). Brain network hierarchy reorganization in Alzheimer’s disease: A resting-state functional magnetic resonance imaging study. *Hum. Brain Mapp.* 43 3498–3507. 10.1002/hbm.25863 35426973 PMC9248302

[B27] Huntenburg J. Bazin P. Margulies D. (2018). Large-scale gradients in human cortical organization. *Trends Cogn. Sci.* 22 21–31. 10.1016/j.tics.2017.11.002 29203085

[B28] Jack C. Bennett D. Blennow K. Carrillo M. Dunn B. Haeberlein S. et al.. (2018). NIA-AA research framework: Toward a biological definition of Alzheimer’s disease. *Alzheimers Dement.* 14 535–562. 10.1016/j.jalz.2018.02.018 29653606 PMC5958625

[B29] Jack C. Knopman D. Jagust W. Petersen R. Weiner M. Aisen P. et al.. (2013). Tracking pathophysiological processes in Alzheimer’s disease: An updated hypothetical model of dynamic biomarkers. *Lancet Neurol.* 12 207–216. 10.1016/S1474-4422(12)70291-0 23332364 PMC3622225

[B30] Jellinger K. Korczyn A. (2018). Are dementia with Lewy bodies and Parkinson’s disease dementia the same disease? *BMC Med.* 16:34. 10.1186/s12916-018-1016-8 29510692 PMC5840831

[B31] Katsumi Y. Zhang J. Chen D. Kamona N. Bunce J. Hutchinson J. et al.. (2023). Correspondence of functional connectivity gradients across human isocortex, cerebellum, and hippocampus. *Commun. Biol.* 6:401. 10.1038/s42003-023-04796-0 37046050 PMC10097701

[B32] Knopman D. Amieva H. Petersen R. Chételat G. Holtzman D. Hyman B. et al.. (2021). Alzheimer disease. *Nat. Rev. Dis. Primers* 7:33. 10.1038/s41572-021-00269-y 33986301 PMC8574196

[B33] Kucikova L. Kalabizadeh H. Motsi K. Rashid S. O’Brien J. Taylor J. et al.. (2024). A systematic literature review of fMRI and EEG resting-state functional connectivity in dementia with Lewy Bodies: Underlying mechanisms, clinical manifestation, and methodological considerations. *Ageing Res. Rev.* 93:102159. 10.1016/j.arr.2023.102159 38056505

[B34] Li X. Bu S. Pang H. Yu H. Zhao M. Wang J. et al.. (2025). Mapping striatal functional gradients and associated gene expression in Parkinson’s disease with continuous cognitive impairment. *NPJ Parkinsons Dis.* 11:138. 10.1038/s41531-025-01002-2 40425604 PMC12117062

[B35] Litvan I. Goldman J. Tröster A. Schmand B. Weintraub D. Petersen R. et al.. (2012). Diagnostic criteria for mild cognitive impairment in Parkinson’s disease: Movement disorder society task force guidelines. *Mov. Disord.* 27 349–356. 10.1002/mds.24893 22275317 PMC3641655

[B36] Liu H. Li Y. Sun Z. Xu X. Yan B. Li Y. et al.. (2025). Altered hemispheres lateralization of brain functional gradients in Alzheimer’s disease. *J. Alzheimers Dis.* 106 139–150. 10.1177/13872877251339761 40400336

[B37] Lowther E. O’Brien J. Firbank M. Blamire A. (2014). Lewy body compared with Alzheimer dementia is associated with decreased functional connectivity in resting state networks. *Psychiatry Res.* 223 192–201. 10.1016/j.pscychresns.2014.06.004 25035300

[B38] Lurie D. Kessler D. Bassett D. Betzel R. Breakspear M. Kheilholz S. et al.. (2020). Questions and controversies in the study of time-varying functional connectivity in resting fMRI. *Netw. Neurosci.* 4 30–69. 10.1162/netn_a_00116 32043043 PMC7006871

[B39] Margulies D. Ghosh S. Goulas A. Falkiewicz M. Huntenburg J. Langs G. et al.. (2016). Situating the default-mode network along a principal gradient of macroscale cortical organization. *Proc. Natl. Acad. Sci. U S A.* 113 12574–12579. 10.1073/pnas.1608282113 27791099 PMC5098630

[B40] Markello R. Misic B. (2021). Comparing spatial null models for brain maps. *Neuroimage* 236:118052. 10.1016/j.neuroimage.2021.118052 33857618

[B41] Markello R. Hansen J. Liu Z. Bazinet V. Shafiei G. Suárez L. et al.. (2022). neuromaps: Structural and functional interpretation of brain maps. *Nat. Methods* 19 1472–1479. 10.1038/s41592-022-01625-w 36203018 PMC9636018

[B42] McKeith I. Boeve B. Dickson D. Halliday G. Taylor J. Weintraub D. et al.. (2017). Diagnosis and management of dementia with Lewy bodies: Fourth consensus report of the DLB Consortium. *Neurology* 89 88–100. 10.1212/WNL.0000000000004058 28592453 PMC5496518

[B43] McKeith I. Ferman T. Thomas A. Blanc F. Boeve B. Fujishiro H. et al.. (2020). Research criteria for the diagnosis of prodromal dementia with Lewy bodies. *Neurology* 94 743–755. 10.1212/WNL.0000000000009323 32241955 PMC7274845

[B44] McKhann G. Knopman D. Chertkow H. Hyman B. Jack C. Kawas C. et al.. (2011). The diagnosis of dementia due to Alzheimer’s disease: Recommendations from the National institute on aging-Alzheimer’s association workgroups on diagnostic guidelines for Alzheimer’s disease. *Alzheimers Dement.* 7 263–269. 10.1016/j.jalz.2011.03.005 21514250 PMC3312024

[B45] Mehraram R. Peraza L. Murphy N. Cromarty R. Graziadio S. O’Brien J. et al.. (2022). Functional and structural brain network correlates of visual hallucinations in Lewy body dementia. *Brain* 145 2190–2205. 10.1093/brain/awac094 35262667 PMC9246710

[B46] Menon V. Uddin L. (2010). Saliency, switching, attention and control: A network model of insula function. *Brain Struct. Funct.* 214 655–667. 10.1007/s00429-010-0262-0 20512370 PMC2899886

[B47] Mesulam M. (1998). From sensation to cognition. *Brain* 121 1013–1052. 10.1093/brain/121.6.1013 9648540

[B48] Murphy K. Fox M. (2017). Towards a consensus regarding global signal regression for resting state functional connectivity MRI. *Neuroimage* 154 169–173. 10.1016/j.neuroimage.2016.11.052 27888059 PMC5489207

[B49] Nicastro N. Mak E. Surendranathan A. Rittman T. Rowe J. O’Brien J. (2021). Altered structural connectivity networks in dementia with lewy bodies. *Brain Imaging Behav.* 15 2445–2453. 10.1007/s11682-020-00444-x 33511557 PMC8500905

[B50] Orlando I. Tan J. Taylor N. Medel V. Wainstein G. Lewis S. et al.. (2025). Dopamine alters functional gradients in Parkinson’s disease. *Imaging Neurosci.* 3:imag_a_00564. 10.1162/imag_a_00564 40800773 PMC12319910

[B51] Ottoy J. Kang M. Tan J. Boone L. Vos de Wael R. Park B. Y. et al.. (2024). Tau follows principal axes of functional and structural brain organization in Alzheimer’s disease. *Nat. Commun.* 15:5031. 10.1038/s41467-024-49300-2 38866759 PMC11169286

[B52] Pang J. Aquino K. Oldehinkel M. Robinson P. Fulcher B. Breakspear M. et al.. (2023). Geometric constraints on human brain function. *Nature* 618 566–574. 10.1038/s41586-023-06098-1 37258669 PMC10266981

[B53] Paquola C. Vos De Wael R. Wagstyl K. Bethlehem R. A. I. Hong S. J. Seidlitz J. et al.. (2019). Microstructural and functional gradients are increasingly dissociated in transmodal cortices. *PLoS Biol*. 17:e3000284. 10.1371/journal.pbio.3000284 31107870 PMC6544318

[B54] Parkes L. Fulcher B. Yücel M. Fornito A. (2018). An evaluation of the efficacy, reliability, and sensitivity of motion correction strategies for resting-state functional MRI. *Neuroimage* 171 415–436. 10.1016/j.neuroimage.2017.12.073 29278773

[B55] Peraza L. Kaiser M. Firbank M. Graziadio S. Bonanni L. Onofrj M. et al.. (2014). fMRI resting state networks and their association with cognitive fluctuations in dementia with Lewy bodies. *Neuroimage Clin.* 4 558–565. 10.1016/j.nicl.2014.03.013 24818081 PMC3984441

[B56] Petersen S. Posner M. (2012). The attention system of the human brain: 20 years after. *Annu. Rev. Neurosci.* 35 73–89. 10.1146/annurev-neuro-062111-150525 22524787 PMC3413263

[B57] Pezzoli S. De Marco M. Zorzi G. Cagnin A. Venneri A. (2021). Functional brain connectivity patterns associated with visual hallucinations in dementia with lewy bodies. *J. Alzheimers Dis. Rep.* 5 311–320. 10.3233/ADR-200288 34113787 PMC8150258

[B58] Postuma R. Iranzo A. Hu M. Högl B. Boeve B. Manni R. et al.. (2019). Risk and predictors of dementia and parkinsonism in idiopathic REM sleep behaviour disorder: A multicentre study. *Brain* 142 744–759. 10.1093/brain/awz030 30789229 PMC6391615

[B59] Power J. Barnes K. Snyder A. Schlaggar B. Petersen S. (2012). Spurious but systematic correlations in functional connectivity MRI networks arise from subject motion. *Neuroimage* 59 2142–2154. 10.1016/j.neuroimage.2011.10.018 22019881 PMC3254728

[B60] Power J. Mitra A. Laumann T. Snyder A. Schlaggar B. Petersen S. (2014). Methods to detect, characterize, and remove motion artifact in resting state fMRI. *Neuroimage* 84 320–341. 10.1016/j.neuroimage.2013.08.048 23994314 PMC3849338

[B61] Preti M. Bolton T. Van De Ville D. (2017). The dynamic functional connectome: State-of-the-art and perspectives. *Neuroimage* 160 41–54. 10.1016/j.neuroimage.2016.12.061 28034766

[B62] Royer J. Paquola C. Valk S. Kirschner M. Hong S. Park B. et al.. (2024). Gradients of brain organization: Smooth sailing from methods development to user community. *Neuroinformatics* 22 623–634. 10.1007/s12021-024-09660-y 38568476

[B63] Schaefer A. Kong R. Gordon E. Laumann T. Zuo X. Holmes A. et al.. (2018). Local-global parcellation of the human cerebral cortex from intrinsic functional connectivity MRI. *Cereb. Cortex* 28 3095–3114. 10.1093/cercor/bhx179 28981612 PMC6095216

[B64] Scheltens P. De Strooper B. Kivipelto M. Holstege H. Chételat G. Teunissen C. et al.. (2021). Alzheimer’s disease. *Lancet* 397 1577–1590. 10.1016/S0140-6736(20)32205-4 33667416 PMC8354300

[B65] Schumacher J. Peraza L. Firbank M. Thomas A. Kaiser M. Gallagher P. et al.. (2018). Functional connectivity in dementia with Lewy bodies: A within- and between-network analysis. *Hum. Brain Mapp.* 39 1118–1129. 10.1002/hbm.23901 29193464 PMC5900719

[B66] Schumacher J. Taylor J. Hamilton C. Firbank M. Donaghy P. Roberts G. et al.. (2021). Functional connectivity in mild cognitive impairment with Lewy bodies. *J. Neurol.* 268 4707–4720. 10.1007/s00415-021-10580-z 33928432 PMC8563567

[B67] Seeley W. Crawford R. Zhou J. Miller B. Greicius M. (2009). Neurodegenerative diseases target large-scale human brain networks. *Neuron* 62 42–52. 10.1016/j.neuron.2009.03.024 19376066 PMC2691647

[B68] Shafiei G. Markello R. Vos de Wael R. Bernhardt B. C. Fulcher B. D. Misic B. (2020). Topographic gradients of intrinsic dynamics across neocortex. *Elife* 9:e62116. 10.7554/eLife.62116 33331819 PMC7771969

[B69] Smallwood J. Bernhardt B. Leech R. Bzdok D. Jefferies E. Margulies D. (2021). The default mode network in cognition: A topographical perspective. *Nat. Rev. Neurosci.* 22 503–513. 10.1038/s41583-021-00474-4 34226715

[B70] Song W. Gong C. Li Y. Yang D. Chen H. Tang L. et al.. (2025). Alzheimer’s disease pathogenesis and the role of APOE ε4 in remodeling functional gradients of the default mode network. *J. Alzheimers Dis*. 108 1726–1737. 10.1177/13872877251389965 41144349

[B71] Sourty M. Thoraval L. Roquet D. Armspach J. Foucher J. Blanc F. (2016). Identifying dynamic functional connectivity changes in dementia with lewy bodies based on product hidden markov models. *Front. Comput. Neurosci*. 10:60. 10.3389/fncom.2016.00060 27445778 PMC4918689

[B72] Sperling R. Aisen P. Beckett L. Bennett D. Craft S. Fagan A. et al.. (2011). Toward defining the preclinical stages of Alzheimer’s disease: Recommendations from the National institute on aging-Alzheimer’s association workgroups on diagnostic guidelines for Alzheimer’s disease. *Alzheimers Dement*. 7 280–292. 10.1016/j.jalz.2011.03.003 21514248 PMC3220946

[B73] Tan J. Kang M. Yeh Y. Bezgin G. Lussier F. Hong S. et al.. (2025). Stepwise connectivity of the entorhinal cortex along connectomic gradients in Alzheimer’s disease. *Brain Commun.* 7:fcaf399. 10.1093/braincomms/fcaf399 41194888 PMC12585350

[B74] Van Essen D. Smith S. Barch D. Behrens T. Yacoub E. Ugurbil K. (2013). The WU-minn human connectome project: An overview. *Neuroimage* 80 62–79. 10.1016/j.neuroimage.2013.05.041 23684880 PMC3724347

[B75] Vázquez-Rodríguez B. Suárez L. Markello R. Shafiei G. Paquola C. Hagmann P. et al.. (2019). Gradients of structure-function tethering across neocortex. *Proc. Natl. Acad. Sci. U S A.* 116 21219–21227. 10.1073/pnas.1903403116 31570622 PMC6800358

[B76] Vidaurre D. Smith S. Woolrich M. (2017). Brain network dynamics are hierarchically organized in time. *Proc. Natl. Acad. Sci. U S A.* 114 12827–12832. 10.1073/pnas.1705120114 29087305 PMC5715736

[B77] Vogel J. Iturria-Medina Y. Strandberg O. Smith R. Levitis E. Evans A. et al.. (2020). Spread of pathological tau proteins through communicating neurons in human Alzheimer’s disease. *Nat. Commun*. 11:2612. 10.1038/s41467-020-15701-2 32457389 PMC7251068

[B78] Vos de Wael R. Benkarim O. Paquola C. Lariviere S. Royer J. Tavakol S. et al.. (2020). BrainSpace: A toolbox for the analysis of macroscale gradients in neuroimaging and connectomics datasets. *Commun. Biol.* 3:103. 10.1038/s42003-020-0794-7 32139786 PMC7058611

[B79] Walker Z. Possin K. Boeve B. Aarsland D. (2015). Lewy body dementias. *Lancet* 386 1683–1697. 10.1016/S0140-6736(15)00462-6 26595642 PMC5792067

[B80] Wang D. Li Z. Zhao K. Chen P. Yang F. Yao H. et al.. (2024). Macroscale gradient dysfunction in Alzheimer’s disease: Patterns with cognition terms and gene expression profiles. *Hum. Brain Mapp.* 45:e70046. 10.1002/hbm.70046 39449114 PMC11502409

[B81] Watson D. Andrews T. (2023). Connectopic mapping techniques do not reflect functional gradients in the brain. *NeuroImage* 277:120228. 10.1016/j.neuroimage.2023.120228 37339700

[B82] Wu J. Ma L. Luo D. Jin Z. Wang L. Wang L. et al.. (2024). Functional and structural gradients reveal atypical hierarchical organization of Parkinson’s disease. *Hum. Brain Mapp.* 45:e26647. 10.1002/hbm.26647 38488448 PMC10941507

[B83] Yao C. Shan Y. Cui B. Chen Z. Bi S. Wang T. et al.. (2025). Hyperconnectivity and connectome gradient dysfunction of cerebello-thalamo-cortical circuitry in Alzheimer’s disease spectrum disorders. *Cerebellum* 24:43. 10.1007/s12311-025-01792-4 39913059

[B84] Yeo B. Krienen F. Sepulcre J. Sabuncu M. Lashkari D. Hollinshead M. et al.. (2011). The organization of the human cerebral cortex estimated by intrinsic functional connectivity. *J. Neurophysiol.* 106 1125–1165. 10.1152/jn.00338.2011 21653723 PMC3174820

[B85] Zarkali A. Thomas G. Hannaway N. Dobreva I. Grant Peters M. Callaghan M. et al.. (2025). Evidence for divergent cortical organisation in Parkinson’s disease and Lewy Body Dementia. *Nat. Commun.* 16:11623. 10.1038/s41467-025-66783-9 41290734 PMC12749319

[B86] Zhou J. Gennatas E. D. Kramer J. Miller B. Seeley W. (2012). Predicting regional neurodegeneration from the healthy brain functional connectome. *Neuron* 73 1216–1227. 10.1016/j.neuron.2012.03.004 22445348 PMC3361461

[B87] Zorzi G. Cecchin D. Bussè C. Perini G. Corbetta M. Cagnin A. (2021). Changes of metabolic connectivity in dementia with lewy bodies with visual hallucinations: A 18F-fluorodeoxyglucose positron emission tomography/magnetic resonance study. *Brain Connect.* 11 518–528. 10.1089/brain.2020.0988 33757301

