# Supplementary material for: Network hierarchy in Alzheimer’s and Lewy body spectrum disorders: functional gradients, cognitive phenotypes, and translational gaps
Source: Front Aging Neurosci. 2026 Aug 10;18:1896407. doi: 10.3389/fnagi.2026.1896407 (PMC13500640; doi:10.3389/fnagi.2026.1896407)
Supplement: Supplementary file 1 [file Data_Sheet_1.docx]

Supplementary Materials

**Supplementary Figure S1. Focused structured narrative evidence audit and operational classification of gradient-related evidence in Alzheimer’s disease and Lewy body spectrum disorders.**

The workflow summarizes literature identification, eligibility screening, and A1–D evidence classification in this non-PRISMA focused narrative review. Nine direct or related gradient studies informed the Alzheimer’s disease synthesis, supporting direct but heterogeneous group-level evidence for metric-specific principal-gradient and default-mode network/transmodal alterations. Fifteen retained Lewy body spectrum/Parkinson’s disease reports informed the LBD/PDD synthesis. One core whole-cortical LBD report contributed both functional-gradient and structural-gradient evidence: no significant group-level differences were observed in the first two static functional gradients, whereas structural-gradient reorganization was reported. Current LBD/PDD evidence therefore identifies FC-derived candidate network targets for direct testing but does not establish a whole-cortical functional-gradient signature.

**Supplementary Tables**

**Table S1. Focused evidence-audit search strategy and eligibility framework**

| Domain | Specification | Operational consequence | Documentation |
| --- | --- | --- | --- |
| Review type | Focused structured narrative review with a transparent evidence audit and candidate-target comparative framework; not a PRISMA-conformant systematic review or meta-analysis. | Evidence was classified to define permissible claims, not pooled for effect estimation. | Sections 1.4 and 3.7 |
| Databases and date | PubMed/MEDLINE was searched from inception to30 April 2026. Google Scholar, forward/backward citation chaining, and recent reviews were used to identify additional eligible reports; a focused update was completed on30 April 2026. | Search transparency is provided without representing the audit as exhaustive. No additional eligible peer-reviewed Tier A1 LBD/PDD report was identified in the focused update. | Section 3.7; Figure S1; final retained sets: 9 AD studies and 15 LBD/PD reports. |
| AD search concept | (Alzheimer* OR "Alzheimer disease" OR "Alzheimer’s disease" OR "MCI due to AD" OR "amnestic mild cognitive impairment" OR "subjective cognitive decline" OR amyloid-positive) AND ("functional gradient*" OR "connectome gradient*" OR "principal gradient*" OR "cortical hierarchy" OR manifold OR "diffusion map" OR connectopic). | Identified direct and related gradient studies summarized in Table 3. MCI or subjective cognitive decline studies were retained only when clinically or biomarker-defined as part of the AD spectrum. | Table 3; Table S2 |
| LBD/PD search concept | ("Lewy body disease" OR DLB OR "dementia with Lewy bodies" OR PDD OR "Parkinson disease dementia" OR PD OR PD-MCI OR MCI-LB) AND ("functional gradient*" OR "structural gradient*" OR "connectome gradient*" OR "cortical gradient*" OR "principal gradient*" OR manifold OR "diffusion map" OR connectopic OR hierarchy). | Identified core LBD, adjacent PD/PDD, and indirect symptom-network evidence summarized in Table 4. | Table 4 |
| Inclusion rules | Peer-reviewed original human imaging studies estimating a gradient, manifold, or comparable low-dimensional connectivity axis were eligible as direct or adjacent evidence. Conventional FC, metabolic-connectivity, or related network studies were retained as Tier C only when phenotype- and network-specific. | Direct evidence must estimate a gradient. Tier C studies can nominate candidate targets for direct testing but cannot establish a functional-gradient effect. | Sections 3.5, 3.7, and 5.5 |
| Exclusion rules | Reviews, editorials, conference abstracts, preprints, non-human studies, studies without original eligible imaging data, and diffuse connectivity findings without phenotype or network specificity. | Prevents ordinary FC literature from being relabelled as direct gradient evidence. | Section 3.7 |
| Operational tiers | A1: direct whole-cortical functional-gradient evidence; A2: direct whole-cortical structural-gradient evidence; B: adjacent gradient evidence; C: indirect FC/connectivity evidence; D: untested theoretical prediction. | A2 does not substitute for A1; C can nominate candidate targets but cannot establish a gradient effect. | Section 3.5; Tables 3–5 |

Note. Search terms were adapted to the syntax of each database; quotation marks denote phrases rather than database-specific field restrictions. Table S1 reports the transparent and operationalized search strategy used for the focused audit.

**Table S2. Methodological, association, and validation audit of Alzheimer’s disease spectrum gradient studies summarized in Table 3**

| Study and evidence scope | Cohort and modality | Gradient method, parcellation/alignment, and metric | Association | Key confounds / robustness | Incremental validity, reliability, and external validation |
| --- | --- | --- | --- | --- | --- |
| Hu et al. (2022) A1: whole-brain functional-gradient evidence | 49 HC, 49 MCI, and 49 AD; rs-fMRI. | Whole-brain functional gradients (G1/G2); regional DMN-associated G2 score alterations, not a canonical G1-compression claim. Parcellation/alignment details not detailed in this concise audit; see primary report. | Logical memory and dementia severity. | Balanced groups; cross-sectional group and correlation analyses. | No reported incremental analysis beyond conventional FC; no external validation or test–retest reported. |
| He et al. (2023) A1: whole-brain functional-gradient evidence | aMCI, AD, and controls; rs-fMRI. | Whole-brain functional gradients and two-dimensional dispersion; G1 suppression and reduced global/module-level dispersion. Parcellation/alignment details not detailed in this concise audit; see primary report. | Cognitive scores. | Whole-brain and module-level analyses; cross-sectional. | No reported incremental analysis beyond conventional FC; no external validation or test–retest reported. |
| Wang et al. (2024) A1: whole-cortical functional-gradient evidence | 809 samples from seven scanners; rs-fMRI. | Whole-cortical principal-gradient analysis; diffusion embedding and template alignment reported; reduced range/variation and regional gradient-score differences. | Meta-analytic cognitive-term and transcriptomic annotation; not an individual-level predictive analysis. | Multi-scanner design; group-level findings were consistent across the reported analysis framework. | No individual-level prediction, reported incremental analysis beyond conventional FC, external validation, or test–retest reported. |
| Liu et al. (2025) A1-related: functional-gradient lateralization | 45 controls, 45 MCI, and 45 AD; rs-fMRI. | Interhemispheric functional-gradient lateralization; reduced DMN/control lateralization. Parcellation/alignment details not detailed in this concise audit; see primary report. | Cognitive association. | Balanced three-group design; cross-sectional. | No reported incremental analysis beyond conventional FC; no external validation or test–retest reported. |
| Ottoy et al. (2024) A1/A2: multimodal functional and structural gradients | TRIAD multimodal cohort; PET, MRI, dMRI, and rs-fMRI. | Functional and structural gradients; subject-specific gradient-space modelling; multiple atlases and Procrustes realignment reported. | Tau burden, reactive microglia, and cognition. | Multimodal design and pathology-network modelling. | No clinical classifier, reported incremental analysis beyond conventional FC, or external diagnostic validation; direct A1/A2 multimodal evidence. |
| He et al. (2024) A1: cortical functional-gradient evidence | 188 cognitively unimpaired older adults with and without Aβ positivity; rs-fMRI. | Large-scale cortical functional gradients and gradient variability; altered hierarchy and reduced variability in Aβ-positive participants. Parcellation/alignment details not detailed in this concise audit; see primary report. | Automated speech features and digit span. | Biomarker-defined preclinical comparison; cross-sectional. | No reported incremental analysis beyond conventional FC; no external validation or test–retest reported. |
| Song et al. (2025) A1-related: DMN functional-gradient analysis | 271 participants stratified by AD diagnosis and APOE ε4; rs-fMRI and plasma Aβ. | DMN functional gradient quantified with BrainSpace; RITG attenuation in APOE ε4 carriers. Alignment details not detailed in this concise audit; see primary report. | Cognition and Aβ–FC–gradient mediation. | Diagnosis-by-genotype design and mediation analysis. | No external validation, test–retest, or reported incremental analysis beyond conventional FC. |
| Tan et al. (2025) Related topology study; not whole-cortical G1 group evidence | AD-spectrum cohort; functional and structural connectome gradients. | Entorhinal stepwise connectivity projected into gradient space; network-propagation/topology result, not whole-cortical G1 group compression. Parcellation/alignment details not detailed in this concise audit; see primary report. | Systems-mechanistic interpretation; not a direct diagnostic endpoint. | Topology/propagation analysis; cross-sectional. | Not designed as an incremental prediction or diagnostic-validation study. |
| Yao et al. (2025) Related subcortical/CTC gradient extension | ADNI-3: 58 AD, 103 MCI, and 85 HC; MRI. | Cerebello-thalamo-cortical connectivity and thalamic/cerebellar gradients; subcortical extension, not whole-cortical principal-gradient evidence. Parcellation/alignment details not detailed in this concise audit; see primary report. | A classification analysis for AD versus controls was reported. | ADNI-derived sample; subcortical extension. | No external validation or reported incremental analysis beyond conventional FC. |

Note. This concise audit focuses on methodological, association, and validation features not detailed in the study-specific synthesis in Table 3. Evidence-tier definitions and search procedures are provided in Table S1 and Section 3.5. “Not reported” means not identified as an explicit analysis in the primary report, not necessarily that the consideration was absent from data processing. A1 = direct whole-cortical functional-gradient evidence; A2 = direct whole-cortical structural-gradient evidence; AD = Alzheimer’s disease; aMCI = amnestic mild cognitive impairment; CTC = cerebello-thalamo-cortical; DMN = default-mode network; HC = healthy controls; MCI = mild cognitive impairment; rs-fMRI = resting-state functional MRI.
